# Propagation of human prostate tissue from induced pluripotent stem cells

**DOI:** 10.1002/sctm.19-0286

**Published:** 2020-03-14

**Authors:** Anastasia C. Hepburn, Emma L. Curry, Mohammad Moad, Rebecca E. Steele, Omar E. Franco, Laura Wilson, Parmveer Singh, Adriana Buskin, Susan E. Crawford, Luke Gaughan, Ian G. Mills, Simon W. Hayward, Craig N. Robson, Rakesh Heer

**Affiliations:** ^1^ Translational and Clinical Research Institute, Newcastle University Centre for Cancer Newcastle University Newcastle upon Tyne UK; ^2^ Acute Internal Medicine University Hospital of North Tees Stockton on Tees UK; ^3^ Prostate Cancer UK/Movember Centre of Excellence for Prostate Cancer, Centre for Cancer Research and Cell Biology Queen's University of Belfast Belfast UK; ^4^ Department of Surgery NorthShore University HealthSystem Evanston Illinois USA; ^5^ Nuffield Department of Surgical Sciences University of Oxford Oxford UK; ^6^ Department of Urology, Freeman Hospital The Newcastle upon Tyne Hospitals NHS Foundation Trust Newcastle upon Tyne UK

**Keywords:** androgen receptor, differentiation, induced pluripotent stem cells, organoids, prostate, prostate cancer, stem cells

## Abstract

Primary culture of human prostate organoids and patient‐derived xenografts is inefficient and has limited access to clinical tissues. This hampers their use for translational study to identify new treatments. To overcome this, we established a complementary approach where rapidly proliferating and easily handled induced pluripotent stem cells enabled the generation of human prostate tissue in vivo and in vitro. By using a coculture technique with inductive urogenital sinus mesenchyme, we comprehensively recapitulated in situ 3D prostate histology, and overcame limitations in the primary culture of human prostate stem, luminal and neuroendocrine cells, as well as the stromal microenvironment. This model now unlocks new opportunities to undertake translational studies of benign and malignant prostate disease.

## Graphical abstract

We demonstrate for the first time that induced pluripotent stem cells (iPSCs) can generate human prostate tissue both in vivo and in vitro, overcoming some significant limitations with patient‐derived xenografts and primary culture to provide a complimentary approach. Furthermore, the in vitro model provides a new platform to create a living biobank of prostate cancer organoids derived from gene editing of iPSCs for preclinical drug testing.

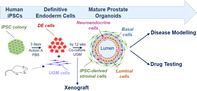

**Significance statement**

Growing cells from prostate cancer biopsies in the laboratory to study mechanisms of disease and to discover new treatments is fraught with difficulties and often not possible. This work establishes a new means to grow “mini 3D prostates” in the laboratory. It shows proof of concept that genetic modifications are possible in this innovative model, which lays the foundations for new preclinical approaches to personalized care previously considered too challenging. Specifically, in future work, one can develop genetically engineered prostate cancers in a dish, tailored to the specific genetic profiles of individual patients, and determine their best response to a range of drug treatments.

## 1 INTRODUCTION

Although the field of treatment‐predictive biomarkers is rapidly developing in prostate cancer, functional tools to undertake preclinical patient‐specific drug testing to more accurately guide outcomes are lacking.^1^ Encouragingly, the capacity to generate in vitro 3D organoid cultures is transforming the study of human diseases.^2^ These structures faithfully mimic in vivo epithelial architecture and present novel opportunities for preclinical studies.^3^, ^4^ However, the widespread adoption of organoid culture in prostate studies is hampered by inherent shortcomings, including limited access to patient samples and the inefficient establishment of cancer organoid cultures. These issues also apply to the other established approach of patient‐derived xenografts (PDXs).^5^ Previously, successful organoid cultures were solely restricted to advanced metastatic tumors^3^; however, recent advances have included the addition of stromal coculture to sustain organoids derived from localized cancers.^6^ In cases where longer‐term cultures are established, an emerging understanding of the substantial genotypic and phenotypic drift that occurs through in vitro culture adaptation restricts their translational value.^7^ Approaches that allow robust isogenic models of cancer are required^8^ and the generation of tissue from pluripotent stem cells appears to be a suitable alternative.

Human embryonic stem cells (ESCs) through coengraftment with rodent urogenital sinus mesenchyme (UGM) can generate prostate tissue in vivo.^9^ However, current in vitro human prostate organoid approaches, from either tissue‐derived cells or ESCs, do not fully recapitulate the full breadth of in situ prostate differentiation as they do not contain neuroendocrine (NE) cells.^4^, ^10^ Of note, emerging data show that NE differentiation drives treatment‐resistant prostate cancer.^11^ Furthermore, alternatives to ESCs would avoid significant ethical and regulatory restrictions and also enable greater access to organoid generation to groups worldwide. The use of induced pluripotent stem cells (iPSCs) is becoming increasingly established in generating tissues from many organs for translational study,^12^ but surprisingly, for the study of the most common male cancer, prostate cancer, the development of such tools remains lagging. We had previously shown the ability to reprogram human prostate cells to provide an easy‐to‐handle and rapidly proliferating source of cells delivering a solution to the problems of limited input from primary biopsies, restrictive ethics and lack of access to patient biopsies.^13^ Thereafter, iPSC lines have also been generated from human fetal prostate fibroblasts, prostate cancer‐associated fibroblasts, and basal prostatic epithelial cells, providing further useful tools to study normal prostate development and prostate disease.^14^, ^15^, ^16^, ^17^

Herein, we demonstrate for the first time that tissue recombinants comprising human iPSCs and rat UGM generated both in vivo xenografts and in vitro prostate organoids that recreated the full breadth in situ prostate epithelial differentiation, including NE cells, as well as the stromal compartment.

## 2 MATERIALS AND METHODS

### 2.1 Patient material

All surgical specimens were collected according to local ethical and regulatory guidelines and included written, informed patient consent (Newcastle REC 2003/11 and Human Tissue Authority License 12 534, Freeman Hospital, Newcastle upon Tyne, United Kingdom). Details of patients from whom iPSC lines were generated are described in Table S1.

### 2.2 Human iPSC generation

iPSC lines were generated from three patients (Table S1). The reprogramming efficiency was 0.02%. For each patient, seven clones were characterized and validated for characteristic ESC marker expression and functional pluripotency in generating all three germ‐layer lineage (see Figure S1 for example of such characterization and validation for patient 13671 clone 1). Human prostate cell culture, characterizations by real‐time polymerase chain reaction (PCR), DNA fingerprinting, karyotyping, immunofluorescence, alkaline phosphatase staining, and assays of pluripotency (embryoid body formation and teratoma formation) were described previously.^13^ Three representative clones from these patients were taken forward and subsequent data were generated.

Pure cultures of 1 × 10^5^ prostate stromal cells seeded on 12‐well plates were transduced using Cytotune 2.0 Sendai Virus reprogramming vectors (KOS, c‐Myc and Klf4, Thermo Fisher Scientific, Waltham, Massachusetts) at a multiplicity of infection of 5‐5‐3 (KOS MOI = 5, hc‐Myc MOI = 5, hKlf4 MOI = 3) as recommended by the manufacturer's instructions, in standard stroma culture medium (RPMI1640 medium with HEPES modification containing 10% fetal bovine serum [FBS, Gibco, Thermo Fisher Scientific], 2 mM L‐glutamine, and 1% penicillin and streptomycin [Sigma‐Aldrich, St. Louis, Missouri]). On day 2, the transduction medium was replaced with fresh standard stroma culture medium. On day 7, cells were seeded onto vitronectin‐coated 12‐well plates at a concentration of 1.5 × 10^4^ cells per well in stroma culture medium. On day 8, medium was replaced with Essential 8 medium (Gibco, Thermo Fisher Scientific) and changed every 48 hours. From day 21, ESC‐like colonies were manually selected based on morphology. Following clonal expansion and characterization, iPSCs were cultured on hESC‐qualified Matrigel (Corning, New York) coated plates in mTeSR1 medium (STEMCELL Technologies, Vancouver, Canada). The medium was changed every 48 hours.

### 2.3 Definitive endoderm induction

To differentiate iPSCs into definitive endoderm (DE) cells, we modified the previously reported DE induction protocol,^18^ with other approaches also showing similar levels of efficiency.^19^ iPSCs were harvested to a single‐cell suspension using Gentle Cell Dissociation Reagent (STEMCELL Technologies) and plated at a density of 2 × 10^6^ cells per well of Matrigel (Corning) coated six‐well plates in mTeSR1 medium (STEMCELL Technologies) containing 1 μg/mL ROCK inhibitor Y‐27632 (STEMCELL Technologies). Cells were incubated at 37°C for 24 hours prior to incubation with DMEM/F12 medium (Sigma‐Aldrich) supplemented with 100 ng/mL Activin A (R&D Systems, Minneapolis, Minnesota). After 24 hours, the media was replaced with DMEM/F12 containing 100 ng/mL Activin A and 0.2% FBS for a further 24 hours. Media was replaced with DMEM/F12 containing 100 ng/mL Activin A and 2% FBS for a final 24 hours incubation.

### 2.4 Tissue recombination grafts of human iPSCs with rat UGM

All animal experiments were performed in accordance with the Institutional Animal Care and Use Committee at North Shore University HealthSystem Research Institute, Evanston, Illinois. Pregnant Sprague‐Dawley rats (Harlan Laboratories Inc, Indianapolis, Indiana) were sacrificed at embryonic day 18. The embryos were isolated and urogenital systems removed. The urogenital sinus (UGS) was separated from the bladder, urethra, Wolffian and Müllerian ducts, and testes or ovaries and incubated in 10 mg/mL trypsin (Sigma‐Aldrich) at 4°C for 90 minutes followed by serial washes with RPMI‐1640 (Sigma‐Aldrich) supplemented with 10% FBS and 1% penicillin and streptomycin (Sigma‐Aldrich). After separation from urogenital sinus epithelium (UGE), based on previous recombination studies,^9^, ^20^ 2.5 × 10^5^ UGM cells were resuspended with 1 × 10^3^, 2 × 10^3^, 1 × 10^4^, and 1 × 10^5^ iPSCs (1:250, 1:125, 1:25, and 1:2.5 ratios, respectively) in 40 μL of rat collagen matrix, plated as a plug and incubated at 37°C overnight in the presence of RPMI‐1640 containing 10% FBS and 1% penicillin and streptomycin. The efficiency of generation of prostate tissue recombinant grafts at 1:125 was 100% (Figure S4).

### 2.5 Athymic nude mouse host xenografting

Male athymic nude mice (Hsd:Athymic Nude‐Foxn1nu; Charles River Laboratories, Wilmington, Massachusetts) aged 10 weeks were used for subrenal capsule grafts. Following castration, a 1‐cm skin incision along the dorsal midline was made, followed by another incision (~6‐8 mm) of the body wall along the line of fat which runs parallel to the spine immediately above the kidney area. Then the kidney was exteriorized and a capsulotomy was made to prepare the subcapsular space for the grafts. Grafts were then placed underneath the renal capsule and maneuvered into various locations along the kidney. Two grafts were placed into each kidney (upper and lower poles), which was then reintroduced back into the mouse. Surgical incisions were closed with suture (body wall) and staples (skin). A testosterone pellet (25 mg) was inserted s.c. into the scruff of the neck.^21^

### 2.6 Xenograft harvest and processing

Hosts were sacrificed 6 weeks after grafting by anesthetic (Penthobarbital) overdose followed by cervical dislocation. Grafts were harvested, and kidneys removed en bloc. Whole kidneys were placed in 10% neutral buffered formalin (Sigma‐Aldrich) for 24 hours. After fixation, kidneys were processed, paraffin was embedded, and sections were cut at 5 μm for Haemotoxylin and Eosin (H&E) staining and immunohistochemistry (IHC).^21^

### 2.7 Coculture of human iPSCs with rat UGM cells

For coculture of UGM and DE cells, chamber slide wells were coated with 40 μL of GFR‐Matrigel (Corning) and set at 37°C for 20 minutes. 1.0 × 10^4^ DE and 3.5 × 10^4^ UGM cells were resuspended in GFR‐Matrigel diluted with DMEM/F12 Ham (1:1, set at 37°C for 30 minutes in the chamber slide wells before addition of DMEM/F12 Ham containing 2% insulin, transferrin, selenium (ITS) (Gibco, Thermo Fisher Scientific) and 10 nM dihydrotestosterone (DHT) (Sigma‐Aldrich). After 7 days, the media was changed to UGM conditioned media collected from whole pieces of UGM incubated in DMEM/F12 Ham containing 2% ITS and 10 nM DHT as previously used for successful in vitro culture of UGS.^22^ The media was further supplemented with 1 μg/mL ROCK inhibitor Y‐27632 (STEMCELL Technologies) and replaced every 48 hours. From 2 weeks, the media was changed to prostate organoid medium.^4^ Wells were harvested 6 weeks onward for histology and RNA extraction. Wells for histology were removed as a Matrigel plug, fixed in 10% formalin overnight, and processed before embedding into paraffin. For RNA extraction, Matrigel was digested by incubation with dispase (STEMCELL Technologies) at 37°C until the gel was completely dissolved. The mixture was gently pipetted to further break up the Matrigel, and transferred to an Eppendorf for centrifugation at 2000 rpm for 5 minutes. The supernatant was removed and the pellet snap frozen in isopentane (Radnor, Pennsylvania) and stored at −80°C.

The efficiency of prostate organoid generation is 100% using an input of 1 × 10^4^ DE cells (with 1:3.5 UGM cells). We were unable to generate prostate organoids using 5 × 10^3^ or less DE cells. Given the inability to utilize <5 × 10^3^ DE cells but achievement of 100% efficiency at the higher density, we did not proceed with exploring further resolution between these densities. At higher ratios, organoids began to merge (Table S2).

### 2.8 Immunohistochemistry

IHC was performed on formalin‐fixed paraffin‐embedded (FFPE) sections (4 μm) that were initially deparaffinized and hydrated. Microwave antigen retrieval was carried out with citrate buffer pH 6 to unmask surface antigens. Endogenous peroxidase activity was removed by blocking with 3% H_2_O_2_ (Sigma‐Aldrich). Sections were then blocked in horse serum (Vector Laboratories, Burlingame, California) and incubated in primary antibody overnight 4°C. The antibodies used were antihuman mitochondria (1:200, Abcam, Cambridge). This is a human specific antibody used in xenographic model research^23^, ^24^, ^25^), p63 (1:50, Leica Biosystems, Wetzlar, Germany), CK8/18 (1:50, BD PharMingen, BD Biosciences, New Jersey), androgen receptor (AR) (1:50, BD PharMingen), NKX3.1 (1:50, AthenaES, Baltimore), prostate‐specific antigen (PSA) (1:25, Biogenex, Fremont, California), and α‐smooth muscle actin (SMA) (1:100, Abcam). Sections were washed and incubated with antirabbit or antimouse secondaries (ImmPRESS HRP Anti‐Rabbit/Anti‐Mouse IgG [Peroxidase] Polymer Detection Kit [Vector Laboratories]). Antibody was detected with DAB solution (ImmPACT DAB Substrate Kit, Vector Laboratories) and counterstained with hematoxylin, dehydrated and mounted using dibutyl phthalate xylene (DPX). Slides were then visualized using Aperio CS2 (Leica Biosystems).

### 2.9 Immunofluorescence

Immunofluorescence was performed on frozen sections (4 μm). Sections were fixed with 4% paraformaldehyde (Alfa Aesar. Haverhill, Massachusetts), permeabilized using 0.1% triton (Sigma‐Aldrich) and blocked in 4% bovine serum albumin (BSA, Sigma‐Aldrich) before incubation with primary antibody overnight at 4°C. The antibodies used were antihuman mitochondria (1:100, Abcam), p63 (1:50, Leica Biosystems), p63 (1:20, BioLegend, San Diego, California used for dual staining), CK8/18 (1:50, BD PharMingen), 34βE12 (1:100, DAKO, Agilent, Santa Clara, California), AR (1:50, BD PharMingen), NKX3.1 (1:50, AthenaES), PSA (1:50, Biogenex), vimentin (1:100, Abcam), α‐SMA (1:100, Abcam), FOXA2 (1:10, R&D Systems), SOX17 (1:20, R&D Systems), chromogranin A (1:100, Abcam), Enolase 2 (1:500, Abcam), synaptophysin (1:10 000, Abcam), and Ki67 (1:20, BD Biosciences). Secondary antibodies Alexa Fluor‐488 (1:100; ab150129; Abcam), Alexa Fluor‐568 (1:100; A‐10042;Thermo Fisher Scientific), and Alexa Fluor‐546 (1:100; A‐11030; Thermo Fisher Scientific) were used to detect the bound unconjugated primary antibody. Sections were washed and mounted using Vectashield with DAPI mountant (Vector Laboratories) before being visualized on a Leica SPE confocal and widefield fluorescence inverted microscope (Leica Biosystems).

Organoids, 3D structures characterized by hollow epithelium, morphologically contain an outer basal layer and inner luminal layer. The percentage of luminal markers was calculated by counting the cells in direct contact with the lumen as the denominator and the numerator was the number of cells that expressed luminal marks CK8/18, NKX3.1, AR, and PSA. Similarly, the percentage of basal marker was calculated by counting cells on the outer basal boundary of the organoid as the denominator and for the numerator we counted the number of these cells that were marked with p63 and 34βE12 (Figure 2K).

### 2.10 RNA extraction, reverse transcription, and real‐time PCR

Total RNA was extracted using Ribozol RNA extraction reagent (Amresco) and reverse transcribed using Moloney murine leukemia virus reverse transcriptase enzyme (Promega, Madison, Wisconsin) according to the manufacturer's instructions. Real‐time PCR (qPCR) was carried out using Platinum SYBR green qPCR SuperMix‐UDG (Thermo Fisher Scientific) in 384‐well clear optical reaction plates using the ABI 7900HT qPCR system (Applied Biosystems, Foster City, California) according to the manufacturer's instructions. Levels of expression were normalized against housekeeping gene GAPDH. Primers were: α‐SMA F: 5′‐CTCACGGAGGCACCCCT‐3′ and R: 5′‐GAAAGTCTCAAACATAATTTG‐3′; FOXA2 F: 5′‐TCCGACTGGAGCAGCTACTATG‐3′ and R: 5′‐CCACGTACGACGACATGTTC‐3′; GAPDH F: 5′‐CGACCACTTTGTCAAGCTCA‐3′ and R: 5′‐GGGTCTTACTCCTTGGAGGC‐3′; HPRT1 F: 5′‐TTGCTTTCCTTGGTCAGGCA‐3′, and R: 5′‐AGCTTGCGACCTTGACCATCT‐3′. Two‐tailed paired *t* test was used to determine statistical significance at a level of *P* < .05.

### 2.11 RNA sequencing analysis

Total RNA was extracted from cells using Ribozol RNA extraction reagent (Amresco, Solon, Ohio) following manufacturer's instructions. RNA‐Seq library construction and sequencing was performed at Otogenetics Corporation (Atlanta, Georgia) according to standard protocols. The resulting RNA‐seq fastq reads were aligned to Hg19 (GRCh37) using STAR^26^ and mapped to genes using HTSeq counts (http://htseq.readthedocs.io/en/master/count.html). Normalized count and differential expression analysis data were generated using DESeq2.^27^ Gene Set Enrichment Analysis (GSEA)^28^, ^29^ was performed on normalized RNA‐seq count data and calculated by permuting genes 1000 times in the GSEA software. Basal and luminal genesets were derived from differential gene expression analysis of iPSCs vs CD49f^+ve^ basal cells or CD26^+ve^ luminal cells isolated from whole human prostates by flow cytometry. All heatmaps were generated using R3.4.2.

### 2.12 Lentiviral transduction

iPSCs were detached from the Matrigel‐coated plates by incubation with dispase (STEMCELL Technologies) for 5‐7 minutes at 37°C. The detached aggregates were then plated onto six‐well Matrigel‐coated plates in mTeSR1 medium with an overall confluency of <40%. After 24 hours, the medium was replaced with the virus‐containing medium (from 293T cells transfected with EF1α‐mWasabi lentiviral vector [Allele Biotech, San Diego, California] and ViraPower lentivirus packaging mix [Thermo Fisher Scientific]) diluted in mTeSR1 medium in the presence of 6 μg/mL polybrene (Merck Millipore, Burlington, Massachusetts). The following day, the virus suspension was replaced with fresh mTeSR1 medium. Five days after transduction, blasticidin was added at final concentration of 1 μg/mL. Selection with blasticidin lasted 12 days with medium and blasticidin changes every 2 days. Fluorescence‐activated cell sorting (FACS) analysis and sorting of EF1α‐mWasabi‐expressing cells was performed on a BD FACSAria III system (BD Biosciences).

## 3 RESULTS

### 3.1 Generation of human iPSC‐derived prostate tissue in vivo

**Figure 1:**
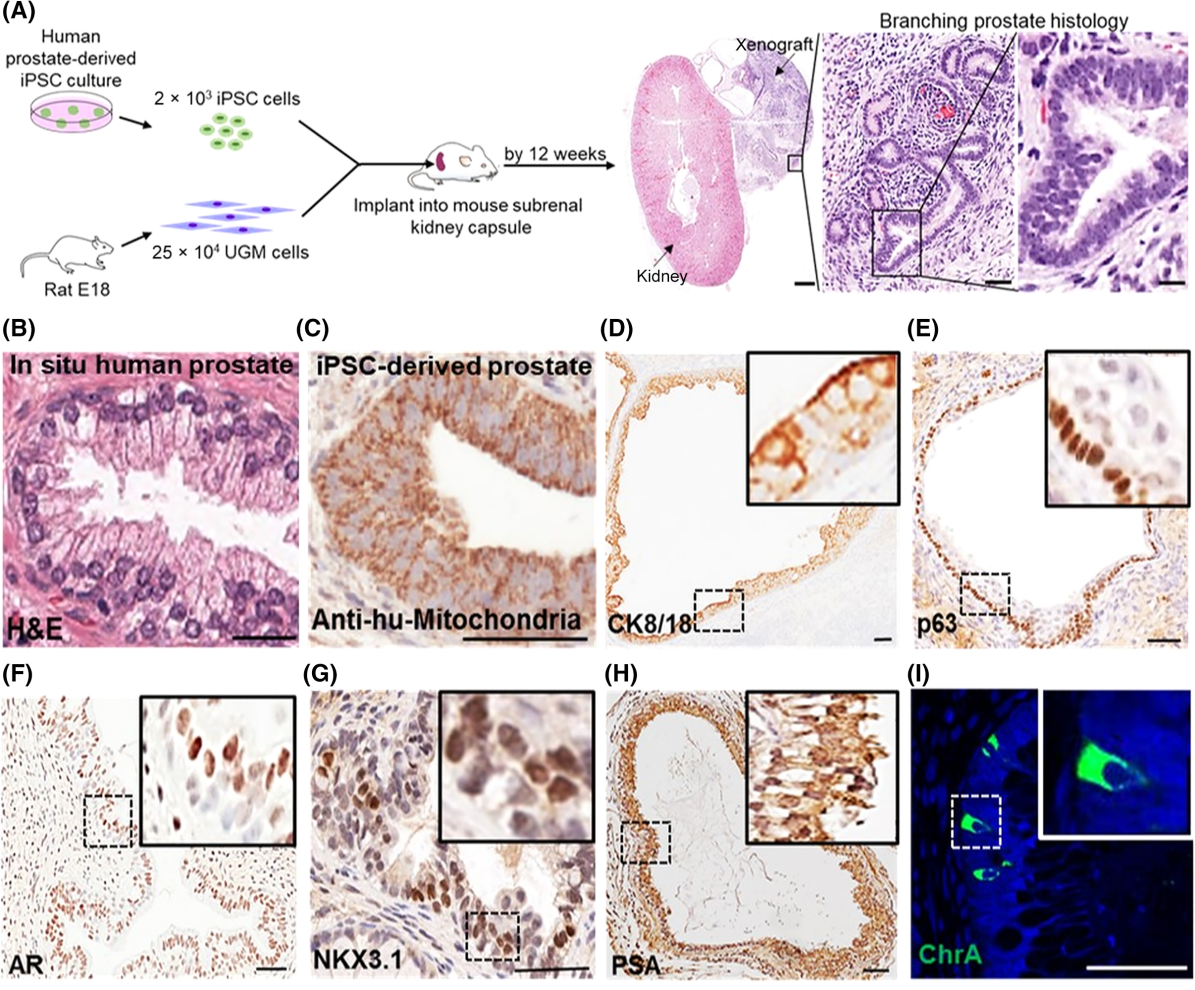
In vivo generation of human prostate tissue. A, Schema overview (n = 12 mice, 4 mice × 3 induced pluripotent stem cell [iPSC] clones). All mice xenografts generated human prostate tissue. Urogenital sinus mesenchyme (UGM) cells injected alone did not develop into glands (data not shown). Scale bars 1 mm (left panel), 50 μm (middle panel), and 15 μm (right panel). B, A reference example of in situ human prostate histology, which is indistinguishable from the xenograft glands shown in A. C, Epithelial cells were confirmed of human origin and not mouse contamination by immunolocalization of antihuman mitochondria (anti‐hu‐mitochondria). D‐E, Luminal and basal epithelium was confirmed by specific differentiation markers CK8/18 and p63, respectively. F‐H, The presence of fully differentiated human prostate was confirmed by expression of androgen receptor, NKX3.1 and prostate‐specific antigen. I, The full breadth of prostate epithelial differentiation was confirmed by the presence of sporadic neuroendocrine cells expressing chromogranin A (ChrA). Scale bar = 50 μm. Nuclei counterstained with DAPI

First, as the tissue of origin used to generate iPSCs can affect subsequent differentiation,^30^ we used a modified integration‐free Sendai virus approach to reprogram human prostate cells^13^ (Figure S1). Reprogramming was confirmed by characteristic ESC morphology and marker expression (Figure S2), and importantly functional pluripotency in generating all three germ‐layer lineages both in vitro and in vivo (Figure S3). To mimic in utero development of the prostate, which is driven by inductive UGM, we undertook subrenal capsule coengraftment of iPSCs with UGM in nude mice (Figure S4).^31^ This resulted in formation of prostatic tissue by 12 weeks (Figure 1), as previously also shown with ESCs.^9^ Grafts comprehensively recreated typical human prostate tissue histology, consisting mainly of glandular structures surrounded by myofibroblasts (Figure 1A,B). The human origin of the epithelial cells was verified by immunolocalization with antihuman mitochondria detection (Figure 1C) and expression of cytokeratins CK8/CK18 on the cell surface and nuclear p63 demonstrated stratification of epithelium into characteristic prostate luminal and basal cells, respectively (Figure 1D,E). AR is an essential driver of prostate differentiation and both nuclear and cytoplasmic expression of the receptor was demonstrated (Figure 1F). Terminal differentiation was confirmed by nuclear localization of prostate specific NKX3.1^32^ and secretory PSA in luminal epithelial cells—again confirming the human nature of the tissue (Figure 1G,H). Critically, Chromogranin A (ChrA) expression identified infrequent NE cells indicating this methodology recapitulated the full breadth of prostate epithelial differentiation (Figure 1I).

### 3.2 In vitro human iPSC‐derived prostate organoids recapitulated the full breadth in situ prostate epithelial differentiation

**Figure 2:**
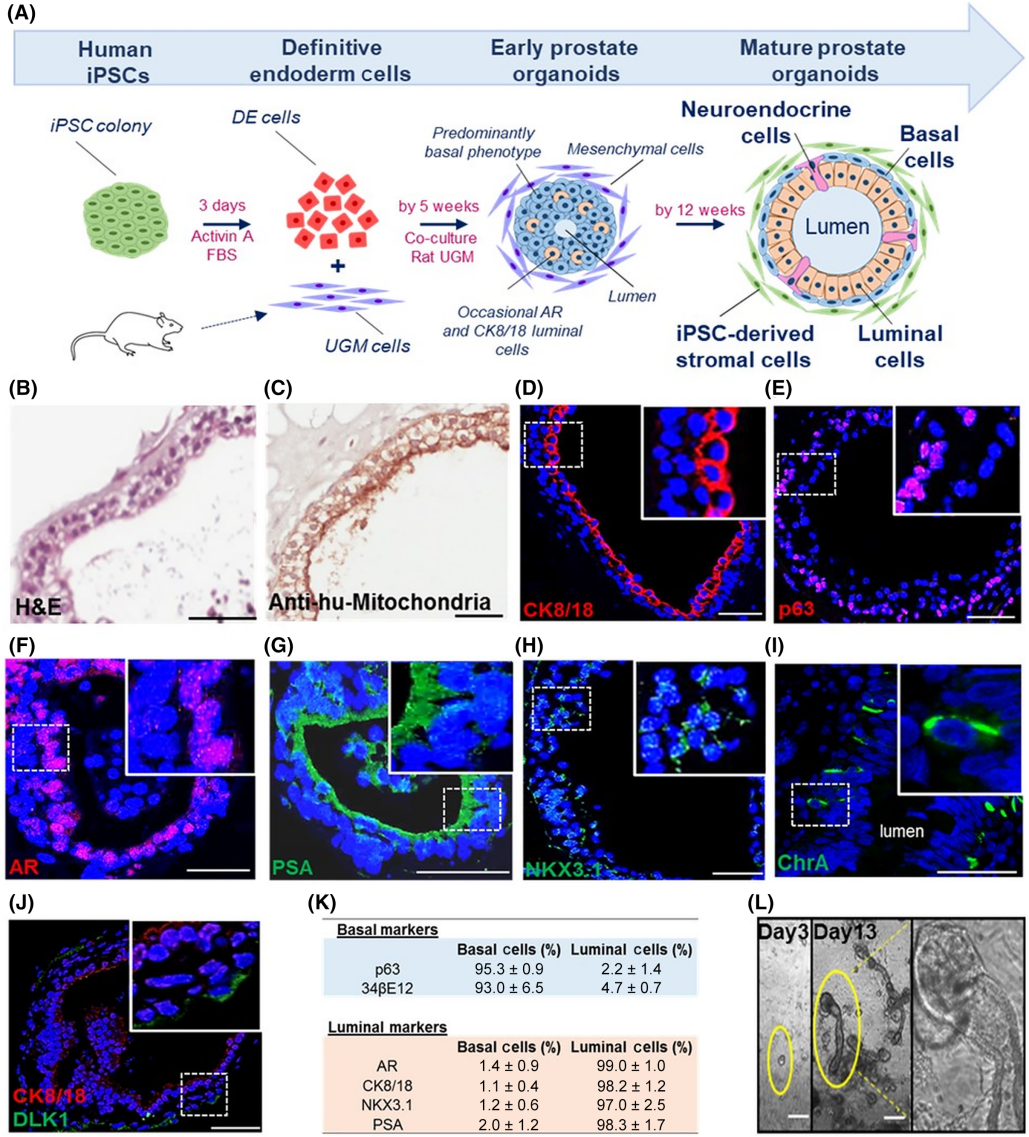
In vitro generation of human prostate organoids. A, Schema outlining the differentiation process (n = 3 induced pluripotent stem cell [iPSC] clones). Briefly, human prostate‐derived iPSCs were differentiated to definitive endoderm (DE) cells using activin A and fetal bovine serum (FBS) over 3 days. Resultant cells were subsequently cocultured with rat urogenital sinus mesenchyme (UGM). At 5 weeks, early prostate organoids were observed having a predominantly basal phenotype while occasionally contained small lumens and expressed luminal markers. Multilayered organoids with large lumens demonstrating classical prostate‐like histology by forming an outer basal and inner luminal layer were observed by 12 weeks. B, Histology of organoids resembled prostate glands. C, Epithelium was identified as human by antihuman mitochondria staining. D, CK8/18 on the cell surface confirmed luminal cells. E, Nuclear p63 confirmed basal cells. F‐H, Androgen receptor (AR), prostate‐specific antigen, and NKX3.1 expression by luminal cells confirmed terminal differentiation. I, Neuroendocrine cells were identified by ChrA expression (0.64 ± 0.21% respectively, n = 7600 cells, n = 12 organoids). J, A subpopulation of basal cells expressed the somatic stem cell marker DLK1 (3.0 ± 1.3%, n = 650 cells, n = 3 experiments). Scale bar = 50 μm. K, Reproducible expression of differentiation markers in mature prostate organoids is shown (n = 183 organoids [164 ± 33 cells/organoid], n = 3 separate experiments; see description of calculations in Materials and Methods). L, Following passage, early clone formation from 1 to 2 cells was observed on day 3, while by day 13 clear canalization was noted of tubular structures associated with dense bud tips reminiscent of tubular branching patterns seen in organogenesis. Scale bar = 25 μm

Because UGM induced prostate differentiation from iPSCs in vivo, we hypothesized it may also direct prostate differentiation in vitro and proceeded to employ a similar 3D coculture methodology (Figure 2A). During embryogenesis, the prostate gland is derived from the endodermal urogenital sinus, thus directing differentiation of iPSCs down an endodermal lineage is likely to increase efficiency of prostatic epithelial differentiation. Accordingly, human prostate‐derived iPSCs were differentiated through a DE intermediary step (Figure S5) using activin A and increasing concentrations of FBS over 3 days, resulting in typical endodermal cobblestone‐like morphology, increased cell size and reduction in the nuclear‐to‐cytoplasmic ratio (Figure S5A).^18^ Enrichment of DE differentiation was confirmed by DE‐specific gene expression (SOX17 and FOXA2 in 75% of cells) (Figure S5B,C). Ten thousand of these cells were subsequently cocultured with 3.5 × 10^4^ rat UGM cells in 3D Matrigel culture. Formation of solid spherical structures was observed at 5 weeks that mimicked embryonic prostate organogenesis characterized by expression of NKX3.1 in cells with a basal phenotype (cytokeratin 34βE12 and p63) (Figure S6A‐C). These structures occasionally contained small lumens associated with luminal marker expression (AR and CK8/18) (Figure S6D,E). Initial solid sphere formation primarily comprised of basal cells is known to proceed the generation of bi‐layered organoids (basal and luminal layers) in both fetal prostate development and also in primary prostate organoid cultures.^4^, ^33^

The ultimate objective was to replicate human prostate histology in vitro, namely multilayered prostate ductal‐acinar epithelium, showing basal and luminal layers, and NE cells with expression of differentiation specific markers. To this end, prostate organoid culture medium,^4^ was applied to cultures from week 2 to support their growth and maintenance. By 12 weeks, multilayered acinar‐like organoids with large lumens (size 65‐455 μm) were observed. These resembled human prostate tissue, demonstrating classical gland histology by forming an outer basal and inner luminal layer (Figure 2A,B). These cells were identified as human by antihuman mitochondria and human specific PSA staining (Figure 2C,G). Expression of CK8/18 and p63 appropriately localized to luminal and basal located epithelial cells, respectively (Figure 2D,E, Figure S7A). Furthermore, luminal epithelial cells expressed nuclear AR and the terminally differentiated nature of the organoids was confirmed by secretory PSA (Figure 2F,G). Previously, NKX3.1, along with AR, were shown to be the essential master regulators of prostate specific differentiation in mice^34^ and we confirmed these expressions in our human prostate organoids (Figure 2H). Sporadic NE cells were again identified by ChrA (Figure 2I) and additionally by Enolase 2 and Synaptophysin expression (Figure S7B). These data demonstrate that similar to the in vivo grafts, the in vitro organoids also faithfully recreated the full breadth of in situ prostate epithelial differentiation and represents a major breakthrough in establishing an easily accessible preclinical model. Of interest, co‐staining of ChrA with Ki67 to determine the proliferative status of NE cells did not reveal coexpression (Figure S8). Additionally, the somatic stem cell enrichment marker DLK1, known to mark basal cells essential for long term maintenance of prostate epithelium, was expressed within a subpopulation of cells (3.0 ± 1.3%, n = 650 cells, n = 3 organoid cultures) (Figure 2J).^35^ The summary statistics are presented, from a total of 183 organoids (n = 3 clones and n = 3 separate experiments), confirming reproducible and appropriate spatially restricted expression of basal and luminal specific markers (Figure 2K). Also, following cellular disaggregation for passage beyond 12 weeks, 3D culture led to branching ductal structures (Figure 2L), therefore fully recapitulating the human prostate histology.

### 3.3 In vitro iPSC‐derived prostate organoids shared gene expression profiles of mature human prostate cells

**Figure 3:**
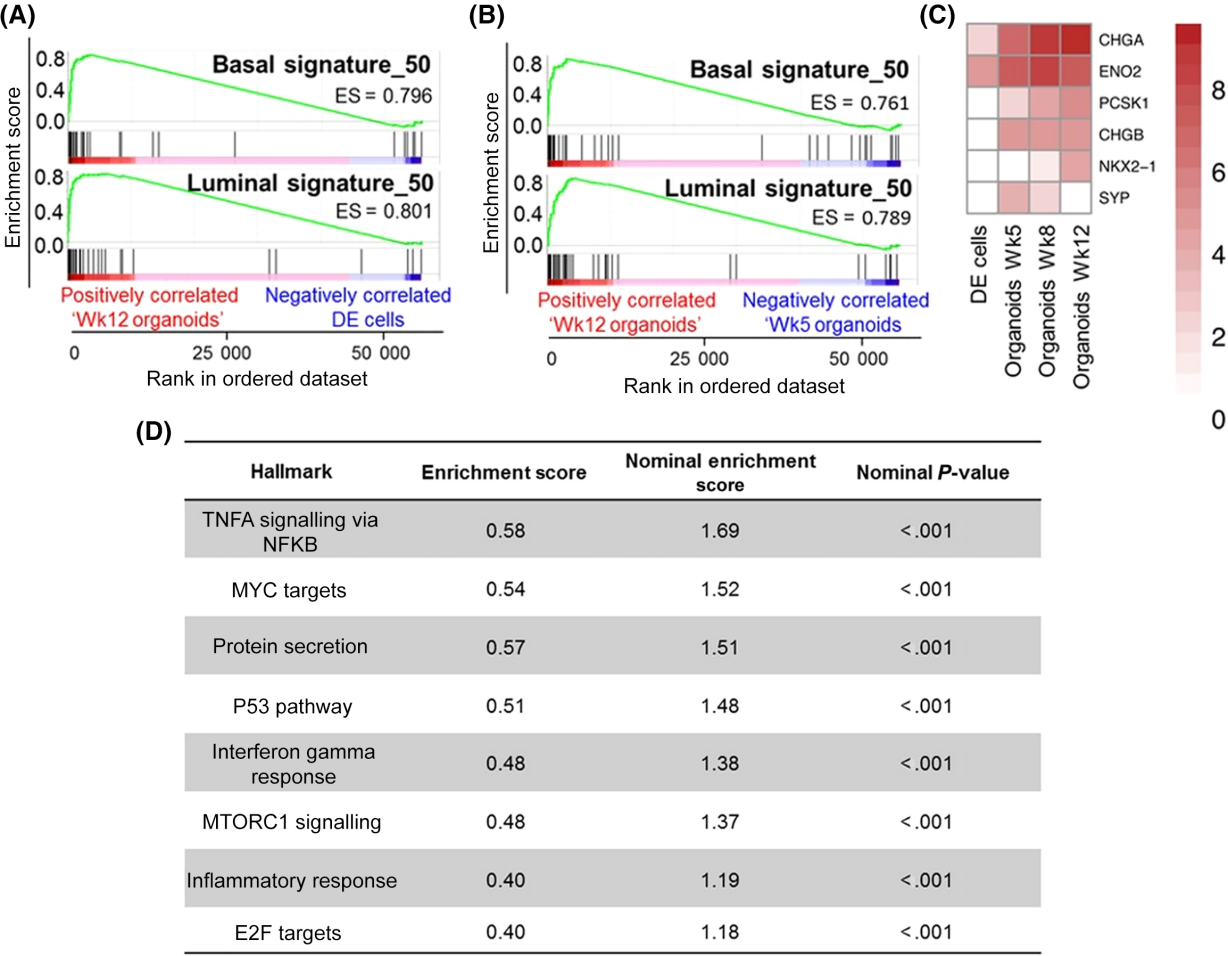
In vitro induced pluripotent stem cell (iPSC)‐derived prostate organoids shared gene expression profiles of mature human prostate cells. A, Gene Set Enrichment Analysis (GSEA) demonstrating enrichment of basal and luminal gene expression in mature organoids (Wk12) in comparison to definitive endoderm (DE) cells (nominal *P*‐value< .001, n = 3 repeats). B, The same as in A, but Wk12 vs Wk5 organoids (nominal *P*‐value <.001). C, Heatmap demonstrating neuroendocrine marker expression. Data is Log2 transformed. D, GSEA Hallmark analysis identified eight statistically significant enriched pathways in mature prostate organoids

Having demonstrated the immunohistological presence of basal, luminal and NE cells, we sought to undertake a broader transcriptomic characterization of the degree of prostate specific differentiation. To dissect the specific ability of the iPSC‐derived prostate organoids to recreate the two main epithelial cell compartments—the basal and luminal cells‐, we compared transcriptomes of iPSCs to primary basal (CD49f^+ve^) and luminal (CD26^+ve^) cells from freshly sorted whole human prostates^4^, ^35^, ^36^ (Figure S9). Comparison of iPSCs to basal cells identified 1775 differentially expressed genes and comparison of iPSCs to luminal cells identified 1213 differentially expressed genes by at least 2‐fold (adj. *P‐*value < .01). Basal and luminal genesets of the top 50 upregulated genes were derived from the differential gene expression analysis of iPSCs vs basal or luminal cells (Tables [Link], [Link]). GSEA confirmed the mature organoids shared the terminally differentiated transcriptomic identities of the benchmark primary adult cells (Figure 3A,B). Mature organoid formation was also characterized by enrichment of NE marker expression (Figure 3C). Additionally, pathway ontology analyses revealed new insights into the mechanisms of differentiation, such as p53, inflammation and Myc related pathways, known to be central in cancer biology and provides focus for future studies (Figure 3D).^37^, ^38^

### 3.4 Prostate iPSCs generated a self‐maintaining stromal compartment in mature prostate organoids

**Figure 4:**
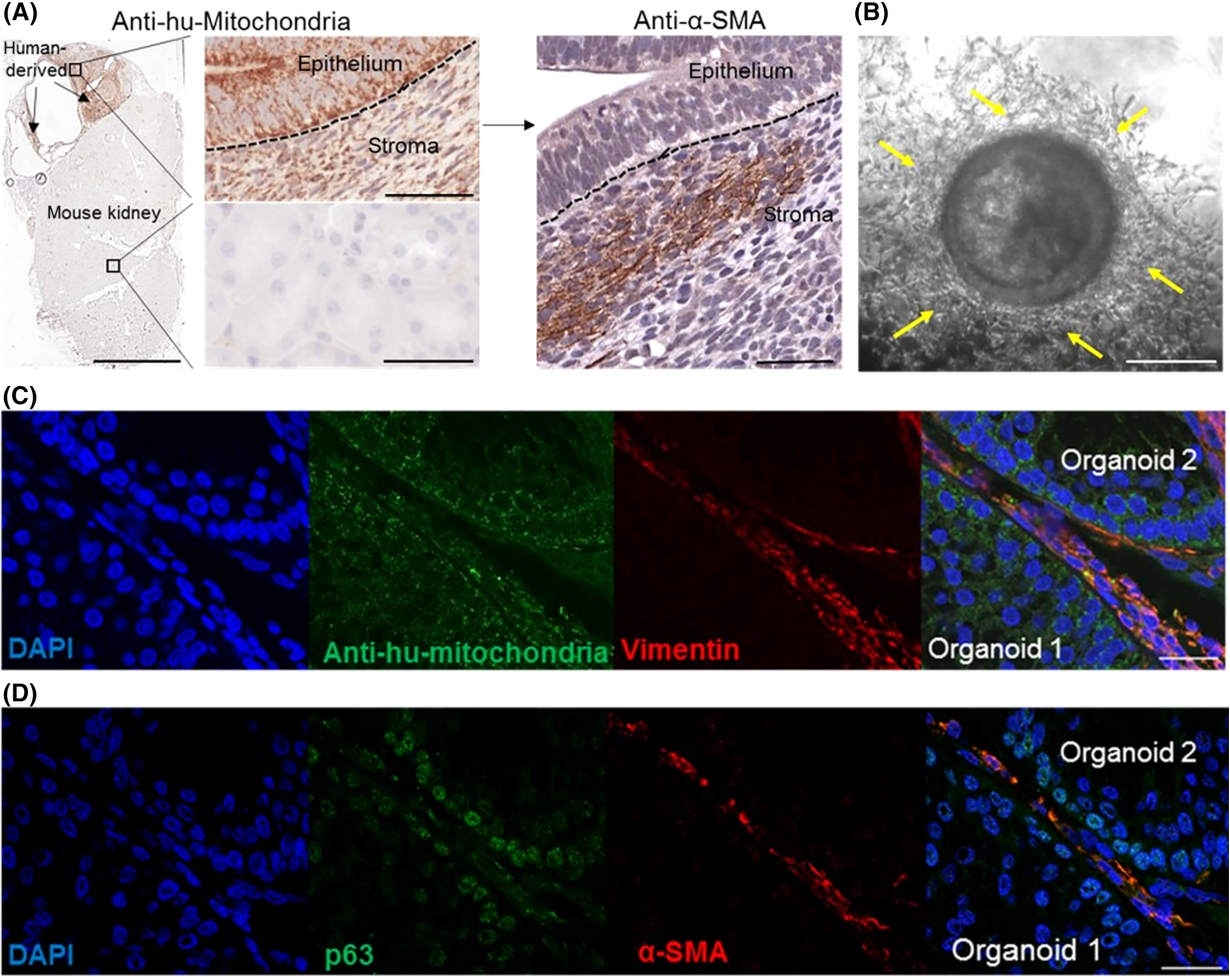
Prostate induced pluripotent stem cells (iPSCs) generated a self‐maintaining stromal compartment in mature prostate organoids. A, Human antimitochondria and α‐smooth muscle actin (SMA) colocalization was seen in stromal cells within areas of the kidney containing the xenograft. Scale bars = 5 mm (left panel) and 50 μm (middle and right panels) (n = 12, 4 mice × 3 iPSC clones). B, Stromal cells surrounded mature prostate organoids. Scale bar = 25 μm. C‐D, Overlapping expressions of vimentin, antihuman mitochondria, α‐SMA and p63 demonstrated capacity of iPSCs to generate the stromal compartment. Scale bar = 25 μm (n = 3 iPSC clones, n = 3 repeats)

In the iPSC‐derived prostate tissue xenografts, glands were surrounded by “nests” of cells with classical stromal morphology (Figure 1A). These were demonstrated to be mesenchymal cells as shown by α‐SMA expression and also of human origin as evidenced by expression analyses of the human specific antimitochondria marker (Figure 4A). These data show that human iPSCs also generated the stromal compartment. In the initial stages of differentiation, the UGM cells, whose inductive properties are transient,^39^ are ultimately replaced by human derived stromal cells, consistent with reciprocal codifferentiation of both epithelial and mesenchymal cells described in utero organogenesis.^31^ We next proceeded to examine whether the iPSCs demonstrated this ability in vitro. Similarly, in mature in vitro‐derived organoids, a self‐maintaining stromal compartment derived from human iPSCs emerged (Figure 4B) when using a DE‐inducing protocol relying on activin A and FBS alone (~75% DE enrichment). This was confirmed by α‐SMA and vimentin colocalization in antihuman mitochondria positive cells surrounding organoids (Figure 4C,D). Of note, when using more efficient methods for DE differentiation (ThermoFisher Scientific kit cat no. A3062601^40^), providing 100% DE differentiated cells determined by FOXA2/SOX17 immunostaining, the cocultures did not yield prostate differentiation (CK positive cells only, data not shown), suggesting that direct stromal differentiation from the iPSCs forms the stromal compartment once the UGM has lost its inductive potential. Further exploration of mesenchymal and epithelial crosstalk in prostate differentiation and disease would be of interest.

## 4 DISCUSSION

In this report, we show for the first time that prostate iPSCs enabled generation of human prostate tissue both in vivo and in vitro. Using UGM‐directed differentiation, human iPSC‐derived prostate tissue comprehensively recapitulated in situ prostate histology and the full breadth of prostate specific epithelial differentiation—including NE cells. The archetypal histology of the human prostate is described by extensive acinar‐tubular branching. Our work has shown that iPSC‐derived organoid cultures also recapitulate this characteristic branching morphology, which is likely to be related to the stromal compartment (derived from the iPSCs) that is known to support branching.^6^

The impact of NE differentiation on prostate cancer progression and driving treatment resistance has recently gained significant attention.^11^, ^41^, ^42^ Although it remains an interesting question whether NE cells can be self‐sustaining in an independent fashion from basal stem cells (DLK1^+^), we have previously demonstrated by in situ lineage tracing using naturally occurring age‐related marks that basal, luminal, and NE cells share a common lineage.^35^, ^43^ However, in nonphysiological states, such as castration, these normal hierarchies can be lost, whereby lineages lose hierarchy dependence, become independent and demonstrate plasticity. Studies have demonstrated emergence of castration‐resistant NKX3.1^+^ luminal cells and induction of NE differentiation markers through plasticity.^11^, ^32^, ^44^ Upon investigation of the proliferative status of NE cells within this report, coexpression of ChrA, and proliferative marker Ki67 was not detected. A lack of such colocalization has also been previously reported and led to the suggestion NE cells do not enter the cell cycle and thus represent a postmitotic subpopulation.^45^, ^46^, ^47^ However, NE cells and Ki67‐expressing cells comprise rare fractions within the normal prostate (1% and 2‐5%, respectively^35^, ^45^) with Ki67 expression typically found in small numbers of basal epithelial cells and sporadically in adjacent stromal cells.^35^ Though we were unable to find coimmunostaining, this does not necessarily exclude existence of this phenomenon.

The establishment of a complementary method to primary culture and PDXs using iPSC‐derived human prostate tissue provides new scope for in vitro disease modeling and drug discovery. Prostate cancer is the second most common male cancer worldwide, accounting for 1.3 million new cases in 2018.^48^ Though genetic testing of tumors to identify actionable mutations is becoming widespread in many cancers, it remains difficult to predict treatment sensitivities to help guide precision medicine.^8^ Ex vivo testing of tumors allows wide scale preclinical testing of multiple treatments.^8^ In this respect, we lay the platform for a radically different preclinical approach where patient‐specific mutations can be introduced into iPSCs to generate bespoke cancer organoids. Our iPSC‐derived approach affords a baseline organoid model that allows the reproducible and targeted generation of specific driver events in the background of patient‐specific germline mutations that are important in prostate carcinogenesis.^49^, ^50^ Previously, it is well established that benign prostate cells can be transformed into prostate cancers^36^, ^51^, ^52^, ^53^, ^54^ and here as a proof of concept we show iPSCs are easily genetically modified by introducing stable fluorescent mWasabi expression using a lentiviral system (Figure S10). Challenges do remain in addressing heterogeneity^55^ and although primary culture remains the preferred solution, it is not an efficient approach for prostate cancer. Inefficiencies associated with tissue accessibility and volume of starting material, as well as in vitro culture drift, are long‐standing difficulties and in this respect reproducible isogenic models are desired. As the number of high frequency genetic drivers in prostate cancer remains low (<3%, n = 1013),^56^ the ability to undertake focused genetic testing for patients and then recapitulate these drivers into isogenic models for functional therapeutic investigations would add to the current translational armory. The generation of a prostate cancer organoid biobank cataloging the mutational landscape of the disease would ultimately form a reference library. We envisage a future where routine genomic testing would define patient‐specific profiles and the library would provide that reference genotype within organoids for new drug testing or previously tested sensitivity to standards of care. This would allow clinicians to tailor treatment options to improve outcomes in cancer patients. Open access to these materials, together with the ease of the tissue generation using the simple protocol described, will result in an immediate impact for many translational researchers worldwide.

Nevertheless, our model already provides insights into tumorigenicity. In the absence of UGM‐mediated initiation of prostate differentiation, uncontrolled differentiation of our iPSCs results in the formation of teratomas emphasizing a role for stromal/mesenchymal cells in the regulation of tumor formation. Quite likely in prostate cancer the normal regulation of epithelial differentiation from the mesenchymal compartment is corrupted by the pathologically affected cancer‐associated fibroblasts. Our model affords an opportunity to dissect this interaction in future studies.

## 5 CONCLUSION

We have generated a complementary method to generate human prostate tissue that opens up new approaches to explore prostate development, homeostasis, and stem cell biology and further lays the foundation for modeling of malignant transformation through gene editing of organoids.

## CONFLICT OF INTEREST

The authors declared no potential conflicts of interest.

## AUTHOR CONTRIBUTIONS

A.H.: collection and assembly of data, data analysis and interpretation, manuscript writing, final approval of manuscript; E.C., M.M., O.F., L.W., P.S., A.B.: collection and assembly of data, data analysis and interpretation, final approval of manuscript; R.S., S.C., L.G., I.M.: data analysis and interpretation, final approval of manuscript; S.H.: provision of study material or patients, collection and assembly of data, data analysis and interpretation, final approval of manuscript; C.R.: conception and design, data analysis and interpretation, final approval of manuscript; R.H.: conception and design, data analysis and interpretation, manuscript writing, final approval of manuscript.

## Supporting information


**Data S1** Supplementary references.

**Figure S1** Reprogramming human prostate cells into iPSCs. We have previously shown that tissue from prostate mesoendodermal lineage is able to generate prostate specific differentiation using an integrative polycistronic lentiviral vector.^1^ Here we show a schematic of the timescale for reprogramming primary prostate fibroblasts to iPSCs using integration‐free Cytotune 2.0 Sendai viral vectors. Micrographs show the change in cell morphology over this period from mesenchymal‐epithelial transition (MET) to appearance of ESC‐like colonies.

**Figure S2** Characterisation of iPSCs. A, ESC‐like morphology of prostate iPSC colony cultured using feeder‐free conditions. Inset, magnified view. Scale bar 10 μm. B, Alkaline phosphatase staining of prostate iPSC colony. Scale bar 10 μm. C, Prostate iPSCs confirmed to possess a diploid 46XY karyotype. D, Immunofluorescence of prostate iPSCs for the expression of specific human ESC surface markers: stage specific embryonic antigen‐4 (SSEA4), tumor rejection antigen (TRA)‐1‐60, TRA‐1‐81, and nuclear transcription factors OCT4 and SOX2. Bottom panel, magnified view. Scale bars 100 μm.

**Figure S3** Pluripotency of iPSCs. A, Immunofluorescence analysis of embryoid bodies derived from prostate iPSCs showing expression of the lineage markers α‐fetoprotein (AFP, endodermal marker, left panel), βIII‐tubulin (ectodermal marker, middle panel) and vimentin (mesodermal marker, right panel). Scale bars 25 μm. Nuclei were counterstained with 4′,6‐diamidino‐2‐phenylindole (blue). B, The absence of stroma/mesenchymal marker expression in the iPSCs confirmed no contamination from non‐reprogrammed prostate stroma cells and subsequent induction of a mesenchymal phenotype was seen only upon differentiation (data represents at least three independent experiments ± SEM). C, Histologic sections of teratoma formed from prostate iPSCs representing all three embryonic germ layers. Scale bars 100 μm, 200 μm and 300 μm.

**Figure S4** Generation of human iPSC‐derived prostate tissue grafts. A, Summary of iPSC and UGM cell densities injected into mice to assess in vivo generation of human prostate tissue. A description of histological observations is included. B, H&E staining demonstrating as the ratio of iPSC:UGM becomes smaller, larger grafts of teratomas are formed. Note for “1 × 10^5^ iPSC + UGM” combination, kidney is out of view due to size of teratoma. Scale bar 2 mm. C, Efficiency of generation of prostate tissue recombinant grafts.

**Figure S5** Formation of definitive endoderm from iPSCs. A, Morphological changes of iPSCs at 72 hours following treatment with Activin A and FBS compared to control (untreated iPSCs) (n = 3 iPSC clones, n = 3 assays per clone). Typical endodermal cobblestone‐like morphology, increased cell size and reduction in the nuclear‐to‐cytoplasmic ratio can be seen. B, Real‐time PCR analysis demonstrating expression of definitive endoderm (DE) specific marker FOXA2 following induction of prostate iPSCs with Activin A and FBS for 72 hours (data represents at least three independent experiments ± SEM, **denotes *P*‐value < .01). C, Immunofluorescence analysis demonstrating expression of DE‐specific markers FOXA2 and SOX17 following treatment of iPSCs with Activin A and FBS for 72 hours. Efficiency of DE differentiation was 75 ± 5%). Inset, magnified view. Scale bar 10 μm.

**Figure S6** Characterisation of early prostate organoids. A, Histology of early organoids demonstrating solid spherical structures (n = 3 iPSC clones, n = 3 repeats). B, Early organoids also predominantly expressed basal marker p63 and luminal transcription factor NKX3.1. C, Predominant expression of basal cytokeratin 34βE12, which was expressed almost uniformly throughout. D, Occasional expression of the transcription factor AR. E, Sparse, expression of luminal cytokeratin CK8/18 was also seen. Scale bars 50 μm. Although infrequently areas of early lumen formation were noted, on the whole we saw amorphous early spheroids consistent with reports of mixed luminal and basal phenotypes before clear differentiation into mature luminal and basal cell histology and restricted expressions of associated differentiation marks. These findings are consistent with the early stages of human fetal prostate development, with an hierarchical pathway of cellular differentiation from basal to luminal cells.^2^, ^3^

**Figure S7** Additional characterisation of human iPSC‐derived prostate organoids. A, Dual CK8/18 and p63 staining confirming luminal and basal cells respectively. B, Sporadic NE cells identified by Enolase 2 (ENO2) and Synaptophysin (SYP) marker expression (0.24 ± 0.02% and 0.32 ± 0.04% respectively, n = 4800 cells, n = 9 organoids). Scale = 50 μm.

**Figure S8** Assessment of the proliferative status of NE cells. Dual staining of proliferative marker Ki67 and NE marker Chromogranin A (ChrA) in iPSC‐derived prostate organoids A, and xenografts B, did not identify coexpression (1.8 ± 0.2% and 0.64 ± 0.21% respectively, n = 7600 cells, n = 12 organoids). Examples of ChrA and Ki67 expressing cells (ChrA^+^ and Ki67^+^ respectively) are noted with arrows. Scale = 50 μm.

**Figure S9** Strategy to isolate basal and luminal cells from whole human prostate. Whole human clinically benign prostates (n = 3) from patients undergoing radical prostatectomy (catheter in urethra) for bladder cancer were processed to isolate basal and luminal epithelial cells for RNA sequencing (as previously described^4^). Briefly, tissue was cut into small chunks and incubated with collagenase. Following trypsinization, single epithelial cells were further enriched by performing MACS EpCAM selection. Samples were stained with basal CD49f and luminal CD26 markers before being FACS sorted. Size gating was applied to enrich for whole cells and doublet discrimination was undertaken to avoid false positive measures. Circular gates identify CD49f^+ve^ basal and CD26^+ve^ luminal cells based on isotype controls.

**Figure S10** Lentivirus‐transduced prostate iPSCs maintained their mWasabi expression upon differentiation. A, Flow cytometry of prostate iPSCs transduced with EF1α‐mWasabi lentivirus (n = 3 repeats). Size gating was applied to enrich for whole cells (P1) and doublet discrimination was undertaken to avoid false positive measures (P2; doublets depicted as red events). 98% of lentivirus‐transduced iPSCs were positive for mWasabi expression (purple events) compared to control untransduced cells. B, Promoter silencing is a known problem in differentiation and we show here using EF1α persistent mWasabi expression—iPSCs (top row) and in differentiated embryoid body outgrowth (bottom row). Phase contrast (left column) fluorescence (middle column), and merged (right column) micrographs are shown. Scale bar = 100 μm.

**Table S1** Details of patients from whom iPSC lines were derived.

**Table S2** Optimization of pluripotent to inductive mesenchymal cell ratio in vitro [Link], [Link]

**Table S3** Differential Gene Expression of iPSCs vs CD49f positive basal cells isolated from whole human prostates by flow cytometry (related to Figure 3 and Figure S4). A list of the genes from RNA sequencing (n = 3).

**Table S4** Differential Gene Expression of iPSCs vs CD26 positive luminal cells isolated from whole human prostates by flow cytometry (related to Figure 3 and Figure S4). A list of the genes from RNA sequencing (n = 3).

**Table S5** List of the top 50 genes upregulated in the DEG analysis to generate “basal” and “luminal” genesets for GSEA.

## Data Availability

The data that support the findings of this study are available from the corresponding author upon reasonable request.
