# Supplementary material for: Propagation of human prostate tissue from induced pluripotent stem cells
Source: Stem Cells Transl Med. 2020 Mar 14;9(7):734–45. doi: 10.1002/sctm.19-0286 (PMC7308643; doi:10.1002/sctm.19-0286)
Supplement: Supplementary file 1 — Data S1 Supplementary references. [file SCT3-9-734-s006.docx]

**Supplemental Data**

**Supplementary References**

1. Moad, M., et al., A novel model of urinary tract differentiation, tissue regeneration, and disease: reprogramming human prostate and bladder cells into induced pluripotent stem cells. Eur Urol, 2013. 64(5): p. 753-61.

2. Bhatia-Gaur, R., et al., Roles for Nkx3.1 in prostate development and cancer. Genes Dev, 1999. 13(8): p. 966-77.

3. Xue, Y., et al., Identification of intermediate cell types by keratin expression in the developing human prostate. Prostate, 1998. 34(4): p. 292-301.

4. Moad, M., et al., Multipotent Basal Stem Cells, Maintained in Localized Proximal Niches, Support Directed Long-Ranging Epithelial Flows in Human Prostates. Cell Rep, 2017. 20(7): p. 1609-1622.
