# Supplementary figures and images for: Propagation of human prostate tissue from induced pluripotent stem cells
Source: Stem Cells Transl Med. 2020 Mar 14;9(7):734–45. doi: 10.1002/sctm.19-0286 (PMC7308643; doi:10.1002/sctm.19-0286)

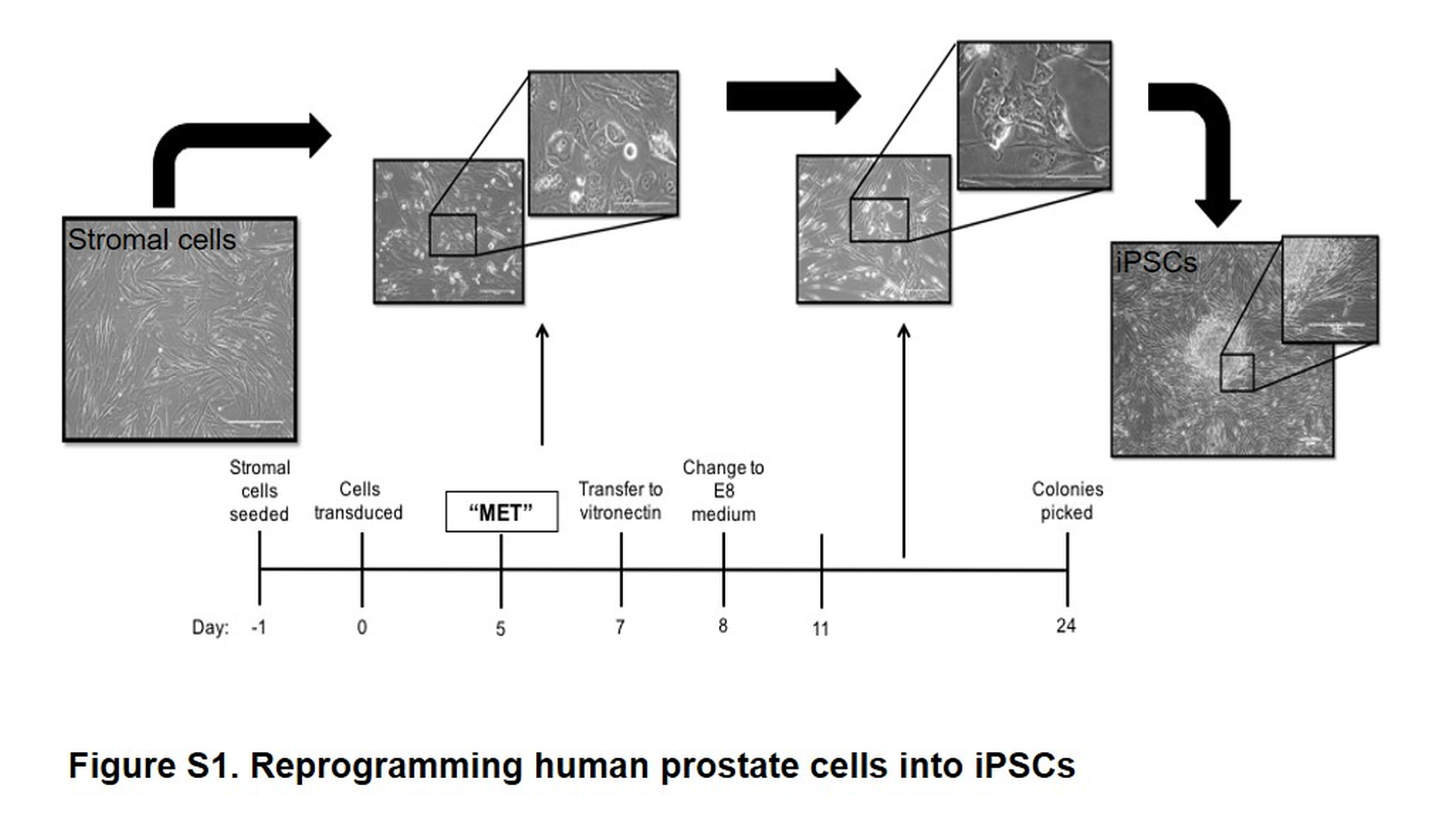

Supplement: Supplementary file 2 — Figure S1 Reprogramming human prostate cells into iPSCs. We have previously shown that tissue from prostate mesoendodermal lineage is able to generate prostate specific differentiation using an integrative polycistronic lentiviral vector.1 Here we show a schematic of the timescale for reprogramming primary prostate fibroblasts to iPSCs using integration‐free Cytotune 2.0 Sendai viral vectors. Micrographs show the change in cell morphology over this period from mesenchymal‐epithelial transition (MET) to appearance of ESC‐like colonies. [file SCT3-9-734-s007.tif]

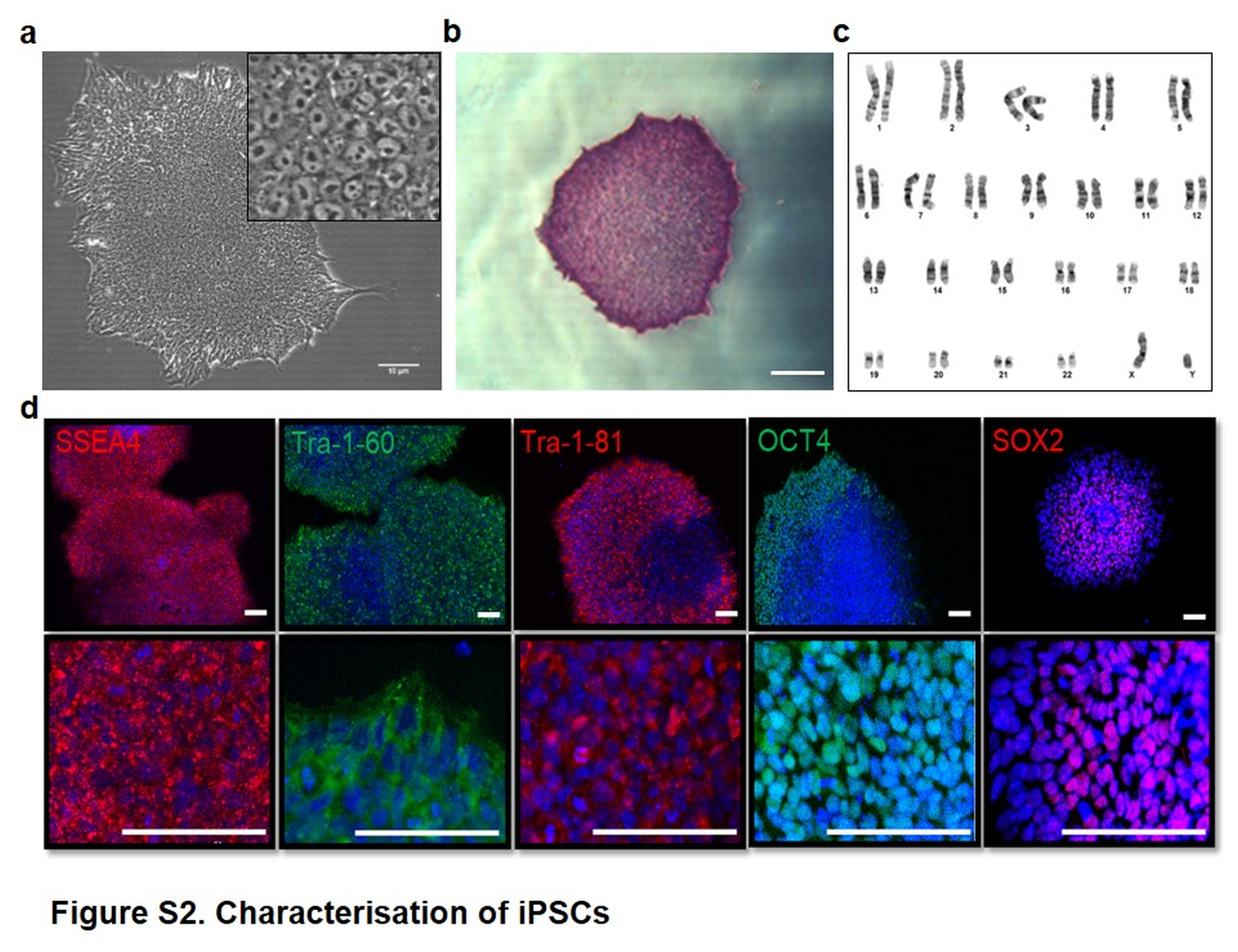

Supplement: Supplementary file 3 — Figure S2 Characterisation of iPSCs. A, ESC‐like morphology of prostate iPSC colony cultured using feeder‐free conditions. Inset, magnified view. Scale bar 10 μm. B, Alkaline phosphatase staining of prostate iPSC colony. Scale bar 10 μm. C, Prostate iPSCs confirmed to possess a diploid 46XY karyotype. D, Immunofluorescence of prostate iPSCs for the expression of specific human ESC surface markers: stage specific embryonic antigen‐4 (SSEA4), tumor rejection antigen (TRA)‐1‐60, TRA‐1‐81, and nuclear transcription factors OCT4 and SOX2. Bottom panel, magnified view. Scale bars 100 μm. [file SCT3-9-734-s008.tif]

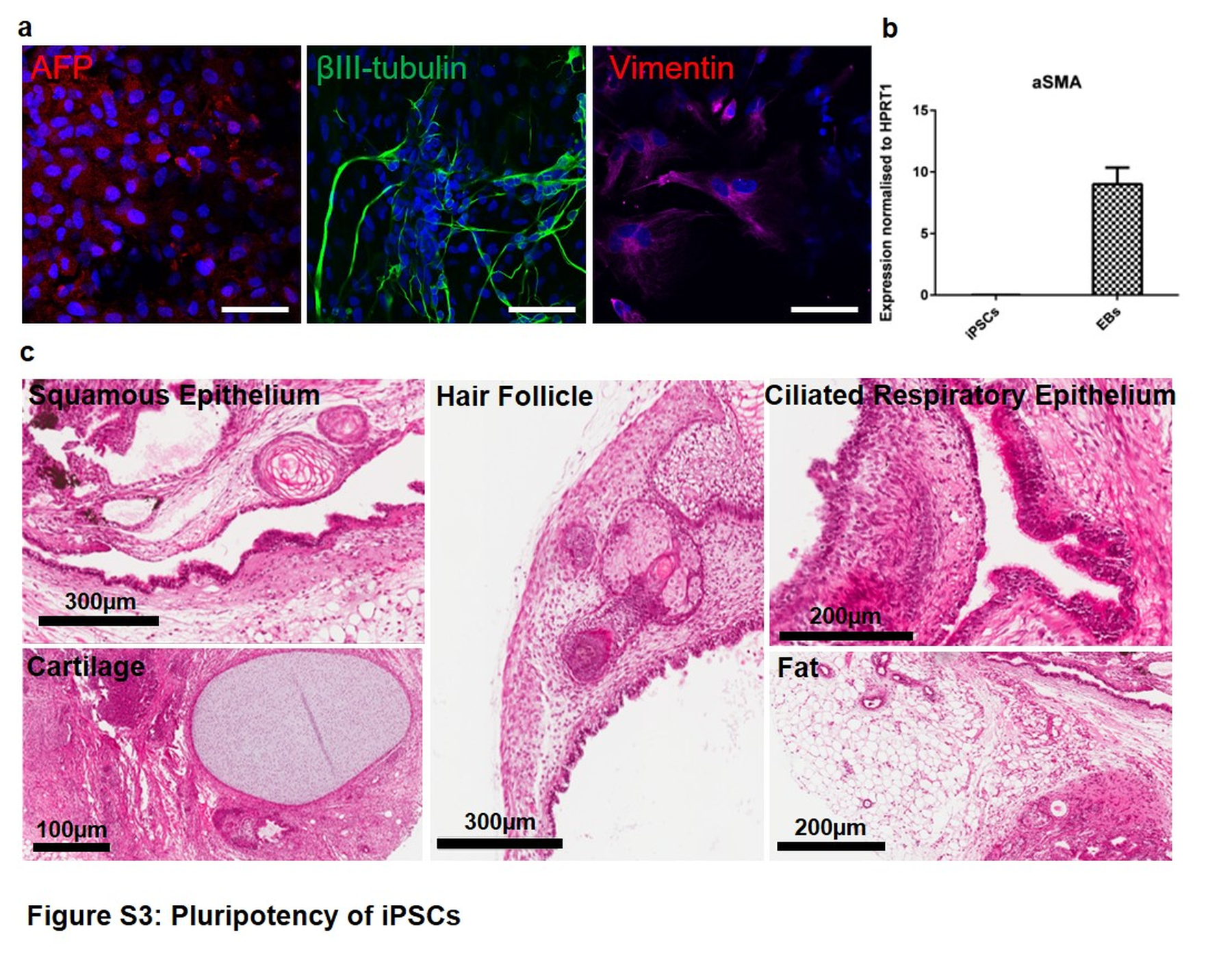

Supplement: Supplementary file 4 — Figure S3 Pluripotency of iPSCs. A, Immunofluorescence analysis of embryoid bodies derived from prostate iPSCs showing expression of the lineage markers α‐fetoprotein (AFP, endodermal marker, left panel), βIII‐tubulin (ectodermal marker, middle panel) and vimentin (mesodermal marker, right panel). Scale bars 25 μm. Nuclei were counterstained with 4′,6‐diamidino‐2‐phenylindole (blue). B, The absence of stroma/mesenchymal marker expression in the iPSCs confirmed no contamination from non‐reprogrammed prostate stroma cells and subsequent induction of a mesenchymal phenotype was seen only upon differentiation (data represents at least three independent experiments ± SEM). C, Histologic sections of teratoma formed from prostate iPSCs representing all three embryonic germ layers. Scale bars 100 μm, 200 μm and 300 μm. [file SCT3-9-734-s009.tif]

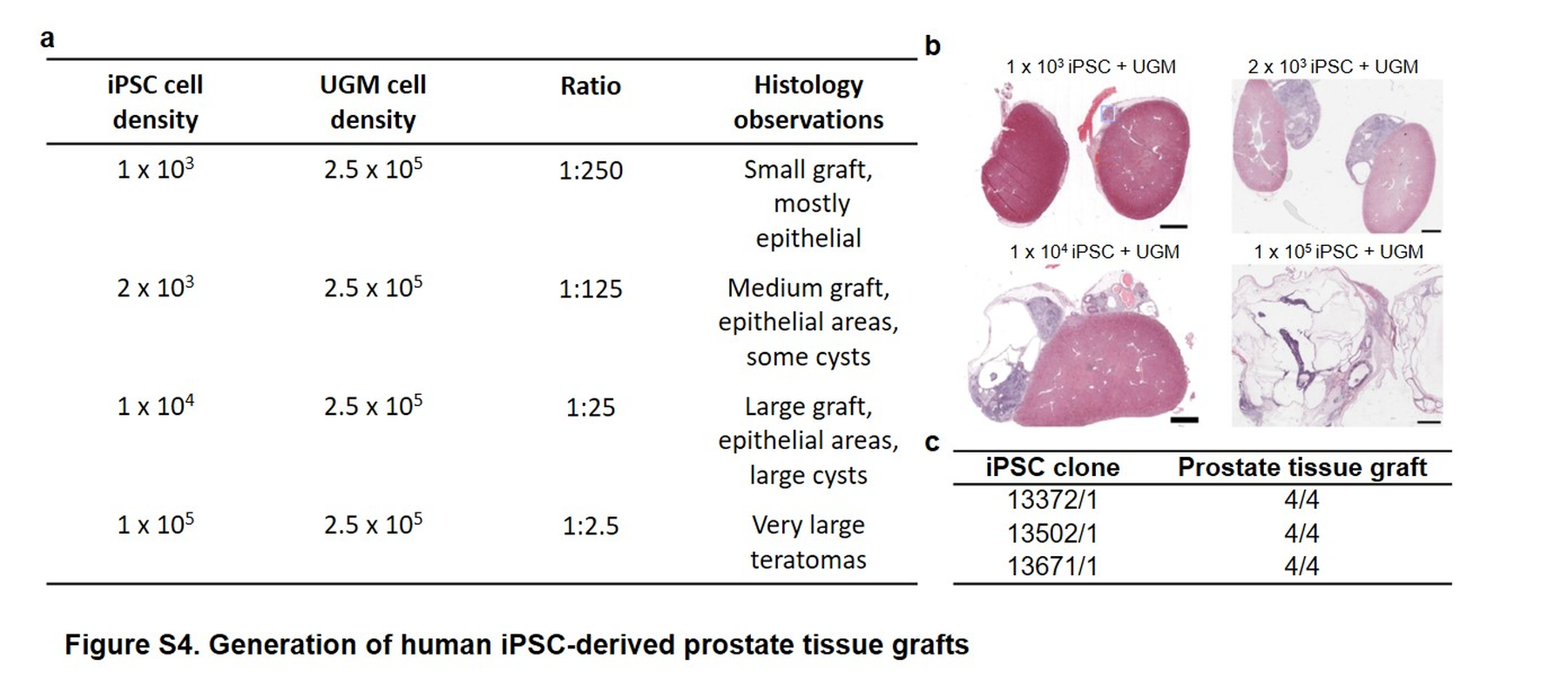

Supplement: Supplementary file 5 — Figure S4 Generation of human iPSC‐derived prostate tissue grafts. A, Summary of iPSC and UGM cell densities injected into mice to assess in vivo generation of human prostate tissue. A description of histological observations is included. B, H&E staining demonstrating as the ratio of iPSC:UGM becomes smaller, larger grafts of teratomas are formed. Note for “1 × 10^5^ iPSC + UGM” combination, kidney is out of view due to size of teratoma. Scale bar 2 mm. C, Efficiency of generation of prostate tissue recombinant grafts. [file SCT3-9-734-s010.tif]

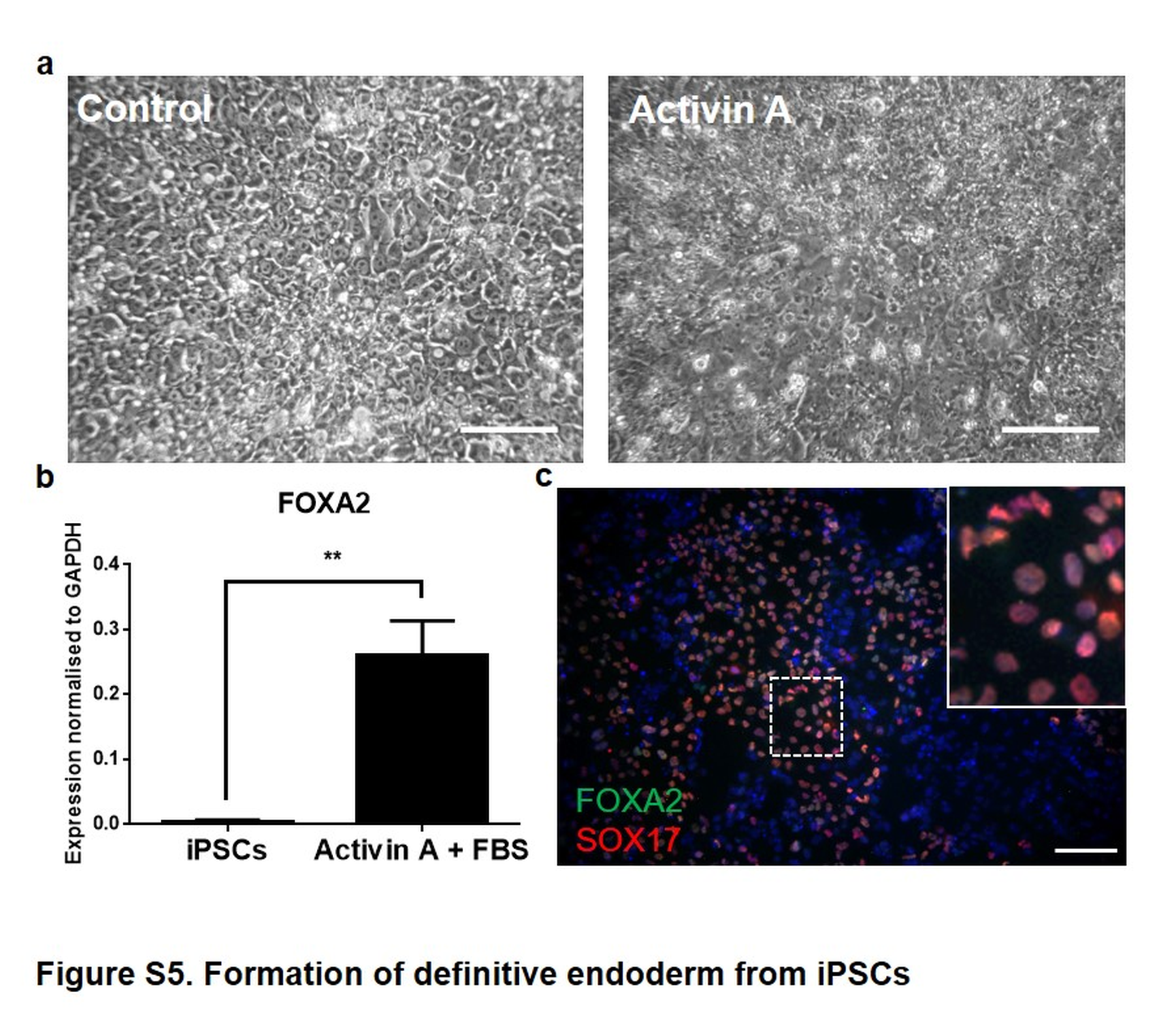

Supplement: Supplementary file 6 — Figure S5 Formation of definitive endoderm from iPSCs. A, Morphological changes of iPSCs at 72 hours following treatment with Activin A and FBS compared to control (untreated iPSCs) (n = 3 iPSC clones, n = 3 assays per clone). Typical endodermal cobblestone‐like morphology, increased cell size and reduction in the nuclear‐to‐cytoplasmic ratio can be seen. B, Real‐time PCR analysis demonstrating expression of definitive endoderm (DE) specific marker FOXA2 following induction of prostate iPSCs with Activin A and FBS for 72 hours (data represents at least three independent experiments ± SEM, **denotes P‐value < .01). C, Immunofluorescence analysis demonstrating expression of DE‐specific markers FOXA2 and SOX17 following treatment of iPSCs with Activin A and FBS for 72 hours. Efficiency of DE differentiation was 75 ± 5%). Inset, magnified view. Scale bar 10 μm. [file SCT3-9-734-s011.tif]

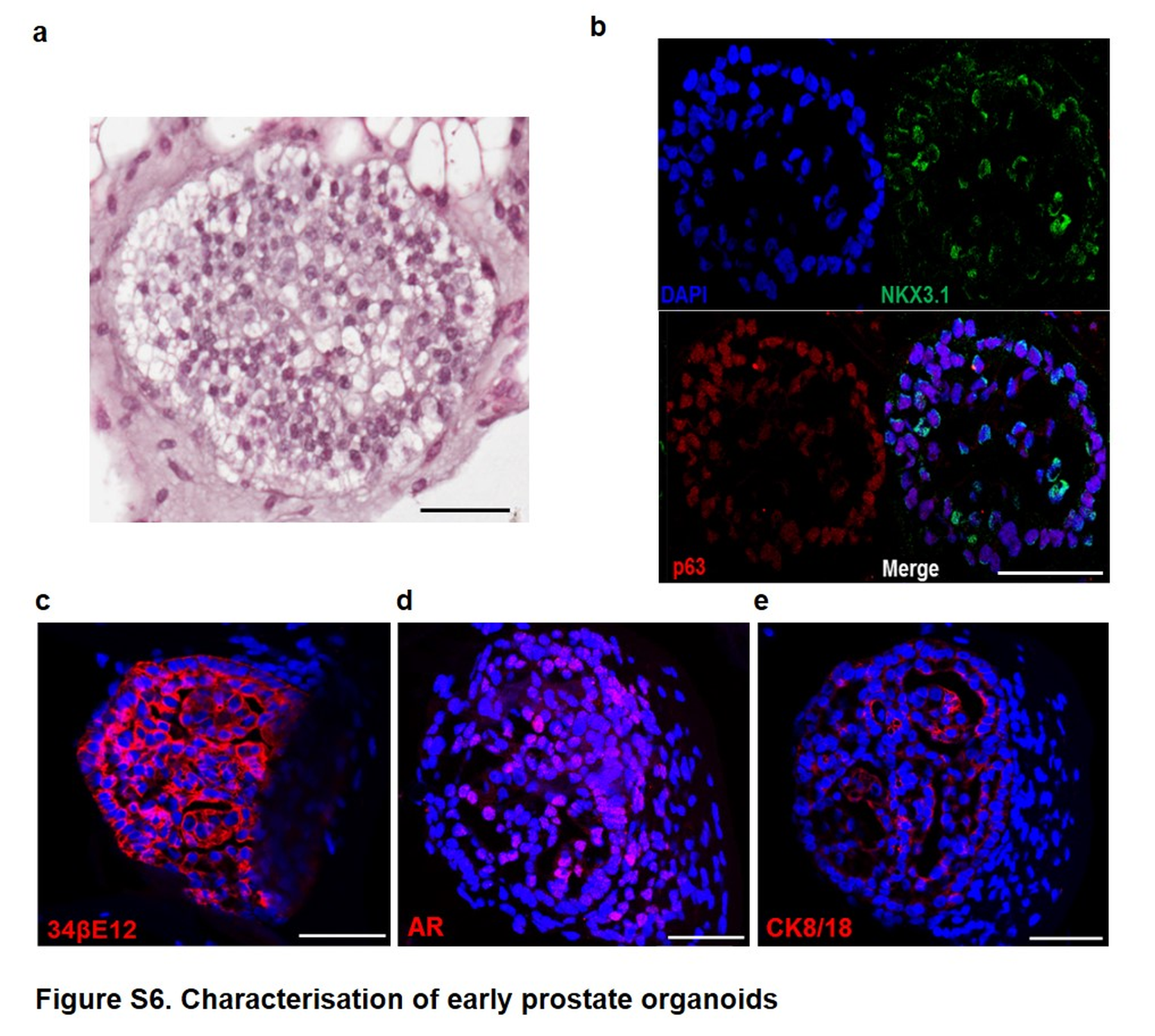

Supplement: Supplementary file 7 — Figure S6 Characterisation of early prostate organoids. A, Histology of early organoids demonstrating solid spherical structures (n = 3 iPSC clones, n = 3 repeats). B, Early organoids also predominantly expressed basal marker p63 and luminal transcription factor NKX3.1. C, Predominant expression of basal cytokeratin 34βE12, which was expressed almost uniformly throughout. D, Occasional expression of the transcription factor AR. E, Sparse, expression of luminal cytokeratin CK8/18 was also seen. Scale bars 50 μm. Although infrequently areas of early lumen formation were noted, on the whole we saw amorphous early spheroids consistent with reports of mixed luminal and basal phenotypes before clear differentiation into mature luminal and basal cell histology and restricted expressions of associated differentiation marks. These findings are consistent with the early stages of human fetal prostate development, with an hierarchical pathway of cellular differentiation from basal to luminal cells.2, 3 [file SCT3-9-734-s012.tif]

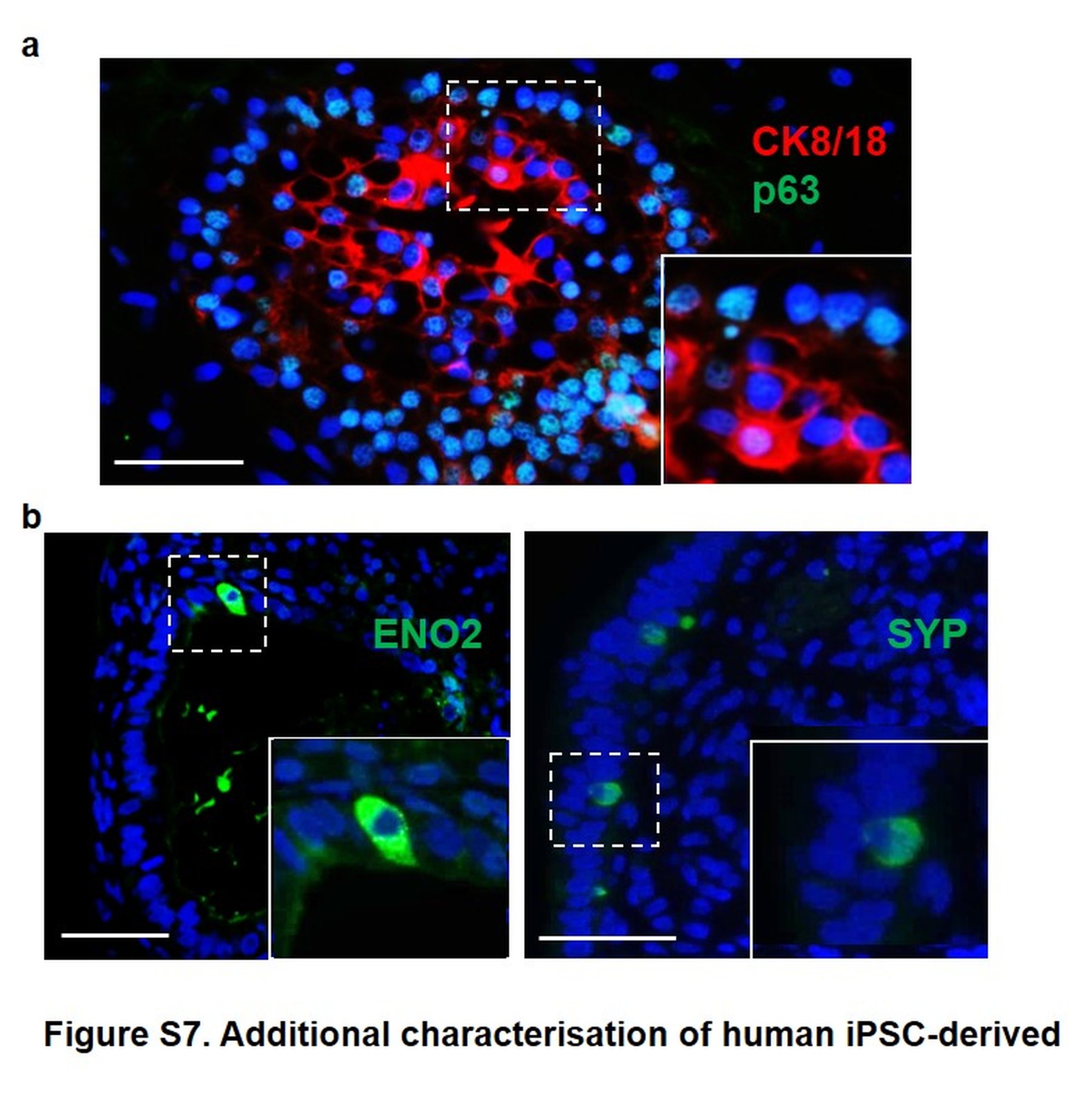

Supplement: Supplementary file 8 — Figure S7 Additional characterisation of human iPSC‐derived prostate organoids. A, Dual CK8/18 and p63 staining confirming luminal and basal cells respectively. B, Sporadic NE cells identified by Enolase 2 (ENO2) and Synaptophysin (SYP) marker expression (0.24 ± 0.02% and 0.32 ± 0.04% respectively, n = 4800 cells, n = 9 organoids). Scale = 50 μm. [file SCT3-9-734-s013.tif]

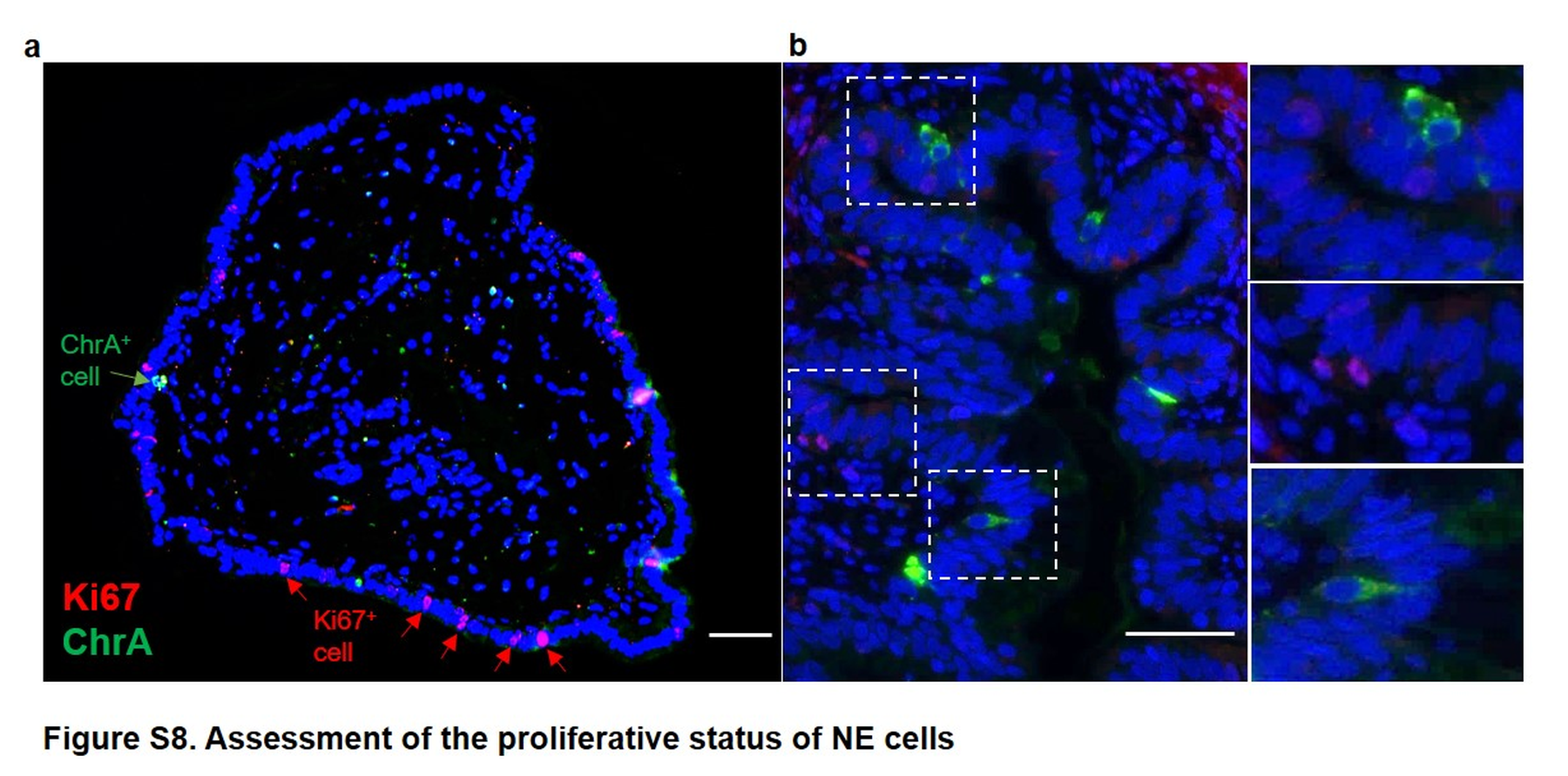

Supplement: Supplementary file 9 — Figure S8 Assessment of the proliferative status of NE cells. Dual staining of proliferative marker Ki67 and NE marker Chromogranin A (ChrA) in iPSC‐derived prostate organoids A, and xenografts B, did not identify coexpression (1.8 ± 0.2% and 0.64 ± 0.21% respectively, n = 7600 cells, n = 12 organoids). Examples of ChrA and Ki67 expressing cells (ChrA+ and Ki67+ respectively) are noted with arrows. Scale = 50 μm. [file SCT3-9-734-s014.tif]

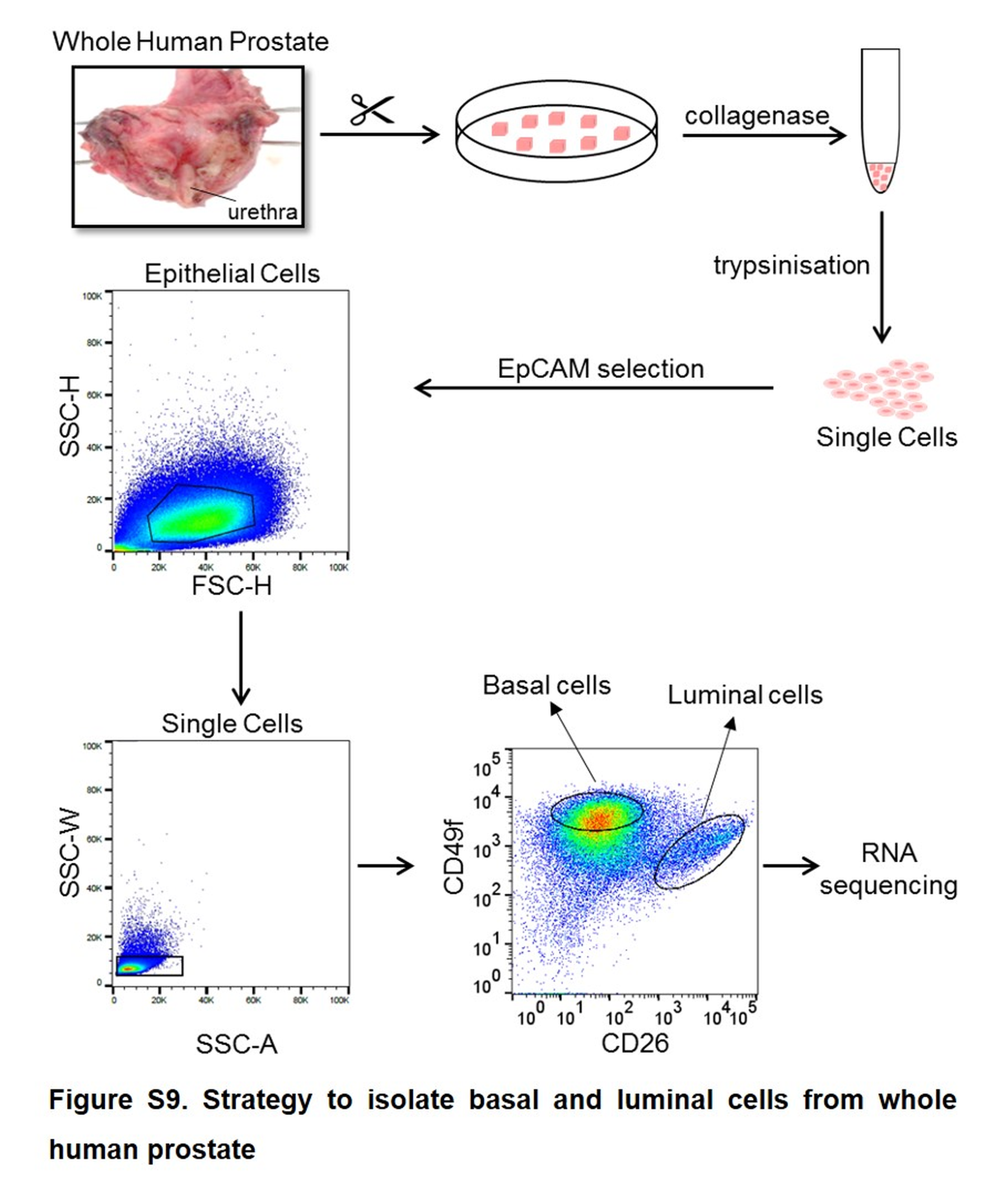

Supplement: Supplementary file 10 — Figure S9 Strategy to isolate basal and luminal cells from whole human prostate. Whole human clinically benign prostates (n = 3) from patients undergoing radical prostatectomy (catheter in urethra) for bladder cancer were processed to isolate basal and luminal epithelial cells for RNA sequencing (as previously described4). Briefly, tissue was cut into small chunks and incubated with collagenase. Following trypsinization, single epithelial cells were further enriched by performing MACS EpCAM selection. Samples were stained with basal CD49f and luminal CD26 markers before being FACS sorted. Size gating was applied to enrich for whole cells and doublet discrimination was undertaken to avoid false positive measures. Circular gates identify CD49f+ve basal and CD26+ve luminal cells based on isotype controls. [file SCT3-9-734-s015.tif]

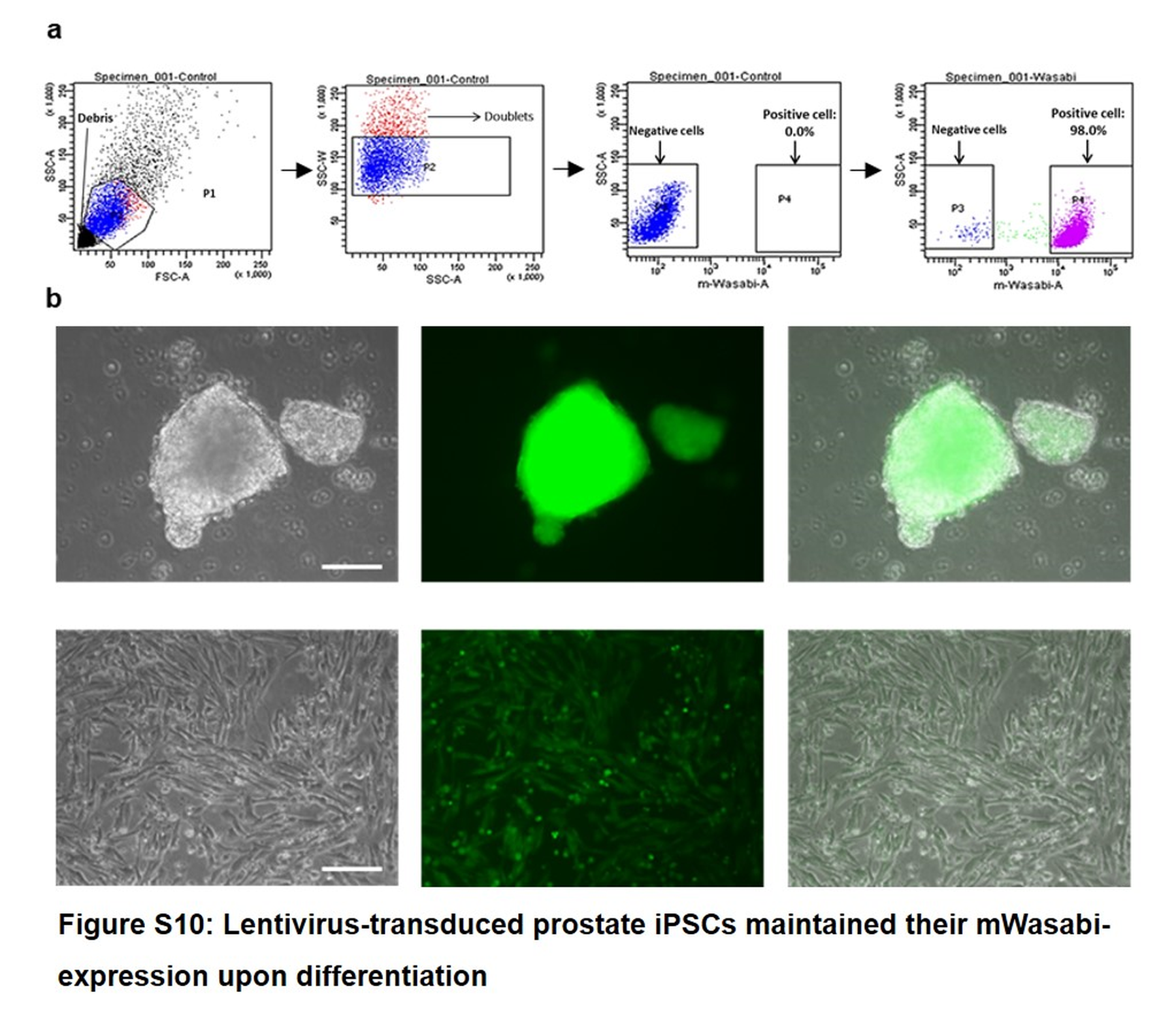

Supplement: Supplementary file 11 — Figure S10 Lentivirus‐transduced prostate iPSCs maintained their mWasabi expression upon differentiation. A, Flow cytometry of prostate iPSCs transduced with EF1α‐mWasabi lentivirus (n = 3 repeats). Size gating was applied to enrich for whole cells (P1) and doublet discrimination was undertaken to avoid false positive measures (P2; doublets depicted as red events). 98% of lentivirus‐transduced iPSCs were positive for mWasabi expression (purple events) compared to control untransduced cells. B, Promoter silencing is a known problem in differentiation and we show here using EF1α persistent mWasabi expression—iPSCs (top row) and in differentiated embryoid body outgrowth (bottom row). Phase contrast (left column) fluorescence (middle column), and merged (right column) micrographs are shown. Scale bar = 100 μm. [file SCT3-9-734-s016.tif]
