# Supplementary material for: Propagation of human prostate tissue from induced pluripotent stem cells
Source: Stem Cells Transl Med. 2020 Mar 14;9(7):734–45. doi: 10.1002/sctm.19-0286 (PMC7308643; doi:10.1002/sctm.19-0286)
Supplement: Supplementary file 12 — Table S1 Details of patients from whom iPSC lines were derived. [file SCT3-9-734-s001.docx]

**Supplementary Table S1. Details of patients from whom iPSC lines were derived.**

| Patient Identifier | Age (yr) | Nature of tissue biopsy |
| --- | --- | --- |
| 13372 | 67 | BPH from TURP |
| 13502 | 78 | BPH from TURP |
| 13671 | 81 | BPH from TURP |

Abbrevations- BPH, benign prostatic hyperplasia; TUPR, transurethral resection of the prostate
