# Supplementary material for: Propagation of human prostate tissue from induced pluripotent stem cells
Source: Stem Cells Transl Med. 2020 Mar 14;9(7):734–45. doi: 10.1002/sctm.19-0286 (PMC7308643; doi:10.1002/sctm.19-0286)
Supplement: Supplementary file 13 — Table S2 Optimization of pluripotent to inductive mesenchymal cell ratio in vitro [Link], [Link] [file SCT3-9-734-s002.docx]

**Supplementary Table S2. Optimisation of DE to inductive mesenchymal cell ratio in vitro**

| **DE cell**  **density** | **Mesenchymal cell**  **density** | **Ratio**  **(DE:mesenchymal)** | **Organoid formation wk 8-12** |
| --- | --- | --- | --- |
| 5 x 10^3^ | 3.5 x 10^4^ | 1:7 | X |
| 1 x 10^4^ | 3.5 x 10^4^ | 1:3.5 | √ |
| 1 x 10^4^ | 5 x 10^4^ | 1:5 | √ |
| 2 x 10^4^ | 7 x 10^4^ | 1:3.5 | √ |
| 1 x 10^4^ | 1 x 10^5^ | 1:10 | √* |
| 1 x 10^4^ | 1.5 x 10^5^ | 1:15 | √* |

* organoids merge
